# The Use of Music in the Treatment and Management of Serious Mental Illness: A Global Scoping Review of the Literature

**DOI:** 10.3389/fpsyg.2021.649840

**Published:** 2021-03-31

**Authors:** Tasha L. Golden, Stacey Springs, Hannah J. Kimmel, Sonakshi Gupta, Alyssa Tiedemann, Clara C. Sandu, Susan Magsamen

**Affiliations:** ^1^Johns Hopkins Medicine, Baltimore, MD, United States; ^2^Department of Health Services, Policy and Practice, Brown University, Providence, RI, United States; ^3^Medical School, University of Michigan, Ann Arbor, MI, United States; ^4^Department of Pharmacy, Birla Institute of Technology and Science, Pilani, India

**Keywords:** mental health, scoping review, music, psychiatric illness, innovative treatment, music therapy, complex intervention, evidence synthesis

## Abstract

Mental and substance use disorders have been identified as the leading cause of global disability, and the global burden of mental illness is concentrated among those experiencing disability due to serious mental illness (SMI). Music has been studied as a support for SMIs for decades, with promising results; however, a lack of synthesized evidence has precluded increased uptake of and access to music-based approaches. The purpose of this scoping review was to identify the types and quantity of research at intersections of music and SMIs, document evidentiary gaps and opportunities, and generate recommendations for improving research and practice. Studies were included if they reported on music's utilization in treating or mitigating symptoms related to five SMIs: schizophrenia, bipolar disorder, generalized anxiety disorder, major depressive disorder, or post-traumatic stress disorder. Eight databases were searched; screening resulted in 349 included studies for data extraction. Schizophrenia was the most studied SMI, with bipolar disorder studied the least. Demographics, settings, and activity details were found to be inconsistently and insufficiently reported; however, listening to recorded music emerged as the most common musical activity, and activity details appeared to have been affected by the conditions under study. RCTs were the predominant study design, and 271 unique measures were utilized across 289 primary studies. Over two-thirds of primary studies (68.5%) reported positive results, with 2.8% reporting worse results than the comparator, and 12% producing indeterminate results. A key finding is that evidence synthesis is precluded by insufficient reporting, widely varied outcomes and measures, and intervention complexity; as a result, widespread changes are necessary to reduce heterogeneity (as feasible), increase replicability and transferability, and improve understandings of mechanisms and causal pathways. To that end, five detailed recommendations are offered to support the sharing and development of information across disciplines.

## Introduction

Mental and substance use disorders have been identified as “the leading cause of disability globally” (Whiteford et al., 2016, n.p.), affecting one in every three to five individuals throughout their lives (see Steel et al., 2014; Vigo et al., 2016). Despite the high prevalence of these disorders, resources and effective treatment remain inconsistently available, with funders and policy makers “fail[ing] to prioritize treatment and care of people with mental illness” (Vigo et al., 2016). Indeed, according to Steel et al. (2014), “substantial evidence indicates the majority of people with mental disorder do not receive specialized services and that global resources allocated to the management of mental disorders is substantially lower than for other chronic health conditions” (p. 490)^1^. In response, health researchers and practitioners have increasingly called for policy changes and additional research to improve treatment, noting that the burden of mental illness will not be reduced without the identification of “more effective ways to provide sustainable mental health services, especially in resource constrained environments” (Whiteford et al., 2013).

In 2020, the need for improved treatment and access skyrocketed as a result of the COVID-19 pandemic. Psychological well-being was undermined by diagnoses and fear of infection (Duan and Zhu, 2020), the necessity of social isolation (Armitage and Nellums, 2020; Brooks et al., 2020), and job loss and subsequent economic hardship (Murthy et al., 2020; Panchal et al., 2020). Health care workers have experienced increased post-traumatic stress disorder (PTSD), stress, insomnia, and depressive symptoms as a result of their work and lack of adequate support (Choudhury et al., 2020; Greenberg, 2020; Spoorthy, 2020). In addition, COVID-19 has disproportionately affected low-income communities and communities of color (Farquharson and Thornton, 2020; Laurencin and McClinton, 2020), exacerbated by the fact that such communities have historically been excluded from access to—and the benefits of—adequate health resources. When combined with the disproportionate prevalence of mental health concerns in these same communities (Coleman et al., 2016, Kilbourne et al., 2018), the current crises make clear that the provision of quality mental health care—as well as policy changes, funding, and innovation to increase access and effectiveness—is an urgent priority for health and health equity.

Given this increasing urgency, it is imperative that mental health researchers and funders prioritize the study of innovative strategies that show promise not only of reducing or eliminating symptoms of mental illness, but also of preventing illness and supporting thriving and resilience. Notably, research and innovation in mental health should be targeted toward SMIs (serious mental illnesses), as “the burden of mental illness is…concentrated among those who experience disability due to SMI” (National Institute of Mental Health, 2019, n.p.). SMIs are defined as “mental, behavioral, or emotional disorder[s] resulting in serious functional impairment, which substantially interferes with or limits one or more major life activities” (National Institute of Mental Health, 2019, n.p.).

To support efforts toward improved and expanded options for addressing SMIs, this scoping review gathers and maps evidence regarding uses of music to treat or mitigate symptoms related to SMI. In doing so, it maps the current research landscape, answering the questions: *What has been researched? How, and with what results? And what barriers and opportunities exist moving forward?*

## Music and Mental Health

For many decades, music has been utilized and studied as a support for mental health—with applications ranging from general mood elevation and stress reduction to clinical interventions designed to treat SMIs. Research indicates that music-based approaches to mental health care can increase patients' likelihood of accessing care^2^ (Schroeder, 2018; Fancourt and Finn, 2019) while reducing its costs (Aalbers et al., 2017). Studies also suggest that mental health treatments that incorporate music may advance health by delivering benefits long associated with arts exposure and participation, such as increased social connectivity (Kreutz, 2014; Welch et al., 2014; Fancourt et al., 2016), additional health-enhancing behaviors (Theorell and Kreutz, 2012; Løkken et al., 2018), and the promotion of identity and resilience (Boggan et al., 2017; Zarobe and Bungay, 2017). Taking place in both clinical and community settings, studies of music's effects on mental health have been conducted within disciplines including psychology (McFerran et al., 2018; Pezzin et al., 2018); neurology (Ventouras et al., 2015; Tan et al., 2016); music therapy (Moe, 2002; Gold et al., 2006); nursing (McCaffrey and Locsin, 2002; Pölkki et al., 2012), dance therapy (Meekums et al., 2015; Campbell, 2020); and psychiatry (Grocke et al., 2008; Grasser et al., 2019), among others.

As one might expect, the many disciplines involved in this work vary widely in how they describe, conduct, and report their studies and practices. As a result, mental health researchers and practitioners have found it difficult to coordinate and synthesize relevant evidence; this has hindered efforts to establish best practices and clinical practice guidelines, develop standardized prescription and treatment models, and promote responsive policies. These limitations have posed ongoing barriers to the testing and scaling of promising strategies that could improve outcomes and access to mental health care.

## Scoping Review

To address these barriers, a scoping review was conducted of the literature regarding uses of music in treatment and symptom mitigation related to SMIs^3^. The purpose of the review was threefold:

- to outline the many practices, interventions, and research processes being undertaken at the intersection(s) of music and SMIs, from all published works;
- to identify evidentiary gaps and opportunities; and
- to offer initial recommendations to support evidence synthesis, improve research practices, and ultimately increase treatment effectiveness and access over time.

## Methods

### Design

This review was conducted utilizing the methodological framework detailed by the Joanna Briggs Institute's (JBI) Methodology for JBI Scoping Reviews (Peters et al., 2020). According to the The Joanna Briggs Institute (2015), the purpose of a scoping review is “to map the key concepts underpinning a research area as well as to clarify working definitions, and/or the conceptual boundaries of a topic” (p. 6). Scoping reviews involve a systematic process of literature searching, screening, and data analysis; the result is a detailed overview of the topic—including who and what have been studied, when and how frequently, research strategies utilized, and preliminary findings. Unlike systematic reviews, scoping reviews do not include a quality assessment of included studies, nor do they generate meta-analyses. Instead, scoping reviews are undertaken regarding emerging topics or knowledge areas, when it is not yet clear what particular questions could or should be addressed via future systematic reviews. Their purpose is to provide a sense of the field's landscape, identifying existing gaps and densities in the evidence.

Over the past decade, heightened interest in researching the mechanistic features of music's effects on mental health has led to an increase in studies; as a result, a scoping review at this point in time advances understanding of the uptake and dissemination of music across disciplines addressing SMIs. Thus, in December 2019, this review's research team searched for similar reviews or protocols using PROSPERO: International Prospective Register of Systematic Reviews, Cochrane Database of Systematic Reviews, BioMed Central Systematic Reviews, and JBI Database of Systematic Reviews and Implementation Reports. None of the resulting reviews had examined ways in which music—including but not limited to “music therapy”—was being utilized in interventions for multiple SMIs.

### Protocol Registration and Reporting

A protocol for this review was developed following the JBI scoping review protocol guidelines (Peters et al., 2020); it was registered in January 2020 with both JBI and the Open Science Foundation (OSF) under the title, “Uses of music in the treatment of serious mental illness: A scoping review.” Reporting of this scoping review was completed in accordance with the PRISMA-ScR Reporting Guidelines.

### Definitions and Classifications

In categorizing music-based interventions represented in included studies, researchers extracted details regarding the musical elements of each intervention—referring to these as the interventions' music-based “activities.” Throughout this article, the term “activities” refers specifically to the music-based elements of the intervention of which they are a part.

Activities were further characterized as “passive,” “active,” or “both.” While levels of felt engagement are likely to vary from participant to participant, these terms allowed the research team to calculate how many activities involved direct/active engagement such as singing or playing an instrument, vs. comparatively passive experiences such as listening to music or watching a performance. This distinction is important, as previous studies have indicated that passive and active engagement in music and other alternative therapies generate varied changes via differing mechanisms (Cosio and Lin, 2018; McPherson et al., 2019; Prakash, 2019).

Activities were additionally categorized as “individual” (involving a single participant either on their own or one-on-one with a facilitator), “group” (involving multiple participants), or “both”—the latter indicating interventions that, for example, alternated between individual and group sessions. Studies regarding the health benefits of social connection suggest that group activities may moderate or mediate effects of music engagement (Holt-Lunstad et al., 2010; Umberson and Karas Montez, 2010; Eisenberger and Cole, 2012), rendering this distinction important.

Finally, most activities involved a therapist, researcher, or other leader who directed or facilitated music-based activities; throughout this article, these individuals are referred to as “facilitator(s).”

### Inclusion and Exclusion Criteria

Because its goal was to illuminate all published research at intersections of music and SMIs, this review did not limit results by date or geographic location. Inclusion criteria were identified using the following PICOS framework:

- Population (P): Humans of all ages being treated for—or assisted in the alleviation of symptoms related to—serious mental illness (SMI), as defined for the purposes of this review by: (1) a diagnosis or suspected diagnosis (verified or self-reported) of schizophrenia, bipolar disorder, MDD, GAD, or PTSD; or (2) inclusion in the experimental group in a study of interventions designed to treat or mitigate symptoms related to these illnesses^4^.
- Intervention (I): Any uses of music when incorporated specifically in the context of a SMI for the purpose of treating or mitigating symptoms related to the SMI. To accomplish the task of mapping all uses of music in this context, the current review drew upon an intentionally broad conceptualization of music-based activities, as differentially conceptualized by varying fields and practitioners. Thus, interventions may involve (but were not limited to) listening to music, playing musical instruments, singing, attending a music-based performance, performing music, dancing^5^, songwriting, or analyzing songs or song lyrics. Uses of music for the promotion of general mental well-being were not included, as these were not pertinent to the SMI classification.
- Comparator (C): Studies comparing music interventions to standard treatments (or treatment as usual (TAU)), studies comparing music interventions to no treatment, and studies comparing music interventions to other non-standard treatments. Studies without a comparator were also eligible for inclusion.
- Outcome (O): All outcomes related to the treatment of SMIs were eligible, such as changes in symptoms, affect, quality of life, functional assessments, academic achievement, social or emotional functioning, delivery of care, and more. Because this review focused on treatment or symptom mitigation related to SMIs, uses of music to *research* SMIs (i.e., outcomes not directly related to changes in symptoms, function, etc.) were excluded.
- Study design (S). All study designs were eligible, including RCTs, pre/post-test designs, qualitative studies, case reports, systematic reviews with and without meta-analyses, *etc*.

Studies that were not available in English (*n* = 24) were excluded from this scoping review. In addition, library closures caused by the COVID-19 pandemic resulted in lack of access to some texts. Great effort was taken by multiple research team members to access all missing texts, using InterLibrary Loan (ILL) requests at multiple research institutions and extensive hand-searches online. Dozens of texts were ultimately located using this strategy; however, 88 studies remained inaccessible, and were ultimately excluded.

### Literature Search

Comprehensive literature searches were conducted by an experienced health sciences librarian in PubMed, the Cochrane Library, PsychINFO, CINAHL, Embase, SCOPUS, RILM, and The Music Periodicals Database. The search strategy was peer-reviewed by an external expert advisor; after recommended modifications were made, it was again peer-reviewed by a second health sciences librarian using the PRESS (Peer Review of Electronic Search Strategies) Checklist (McGowan et al., 2016). Once searches were conducted, librarians exported results from each database into EndNote, de-duplicated them, and uploaded them into the Covidence platform (https://www.covidence.org/home) for screening by the research team.

### Data Collection and Analysis

Title and abstract screening was conducted in Covidence by blinded pairs of research team members. Full-text screening was conducted by the research team, with data extraction completed in a shared Google Sheets file.

### Critical Appraisal

As noted above, scoping reviews differ from systematic reviews in that their objective is to provide “an overview of existing evidence regardless of methodological quality or risk of bias” (Tricco et al., 2018). In keeping with the aims of this scoping review, included sources were not critically appraised.

## Results

### Numerical Summary

This scoping review's literature search identified 11,967 studies for potential inclusion. Following title/abstract screening and full-text screening, 349 articles were found to meet the inclusion criteria; they have been included in the following analysis. See Data Sheet 1 for a complete list of articles.

### Analysis

Data were analyzed by diagnosis, population, musical activity, study design, variables or outcomes measured, and findings. Studies indicated considerable variations in purposes, intervention strategies, populations, settings, facilitator identities/roles, measurements, outcomes, and reported details. Because secondary studies incorporated multiple primary studies, some of which appeared separately in this scoping review, secondary studies were excluded from analyses that sought frequency counts of factors such as demographics, setting, facilitator details, *etc*. This prevented duplicative reporting.

### Sample Size and Populations

Of the 289 primary studies, 10 (3.4%) did not record a study sample size. Among the remaining studies, sample size ranged from one to 1,000 participants, with a mean sample size of 48.8 and a median of 20.

Demographic reporting varied widely. Of the 289 primary studies, 19 (6.6%) did not report on gender. Of the remaining studies, 50 (17.3%) involved female participants, and 40 (13.8%) involved male participants. One hundred seventy-nine studies (61.9%) were “mixed group,” meaning that multiple genders were included (breakdown was not consistently reported).

Race and ethnicity were inconsistently reported, with 221 primary studies (76.5%) failing to report race or ethnicity at all. Of those reporting this variable, the majority included multiple races, although the breakdown was inconsistently reported.

Age was also inconsistently reported, with 124 primary studies (42.9%) failing to report minimum participant age, and 131 (45.3%) failing to report maximum age. Thirty-one primary studies (10.7%) reported the numeric age of a single participant. Across all studies, a range in age from 3 to 100 is represented, with apparent density between the ages of 18 and 60.

Apart from demographics, data extraction included populations/groups under study, such as “veterans” (Wilbur et al., 2015), “inpatients with schizophrenia” (He et al., 2018), and survivors of domestic violence (Hernández-Ruiz, 2005). Such groups were initially documented verbatim, and later coded by researchers according to the list in Table 1, selecting the most specific population group discernible from the full text^6^. Given the complexity of research at the intersections of music and SMIs, it is likely that targeted interventions (for particular groups, settings) would support the development and synthesis of evidence and best practices.

**Table 1:** Population.

**Population**	***N***	**%**
Inpatients (general)	65	22.5
Person(s) with SMI(s)	62	21.4
Patients (location unspecified)	52	18.0
Other	18	6.2
Veterans and/or Military Personnel	18	6.2
Outpatients (general)	15	5.2
Adolescent(s)	14	4.8
Elderly Individual(s)	10	3.5
Child(ren)	11	3.8
Refugees	9	3.1
Survivor(s) (general)	6	2.1
Child(ren) and Adolescents	4	3.8
Prisoners	3	1.0
IPV/DV Survivors	2	0.7
Total	289	100.0

### Studies Over Time

**Figure 1:**
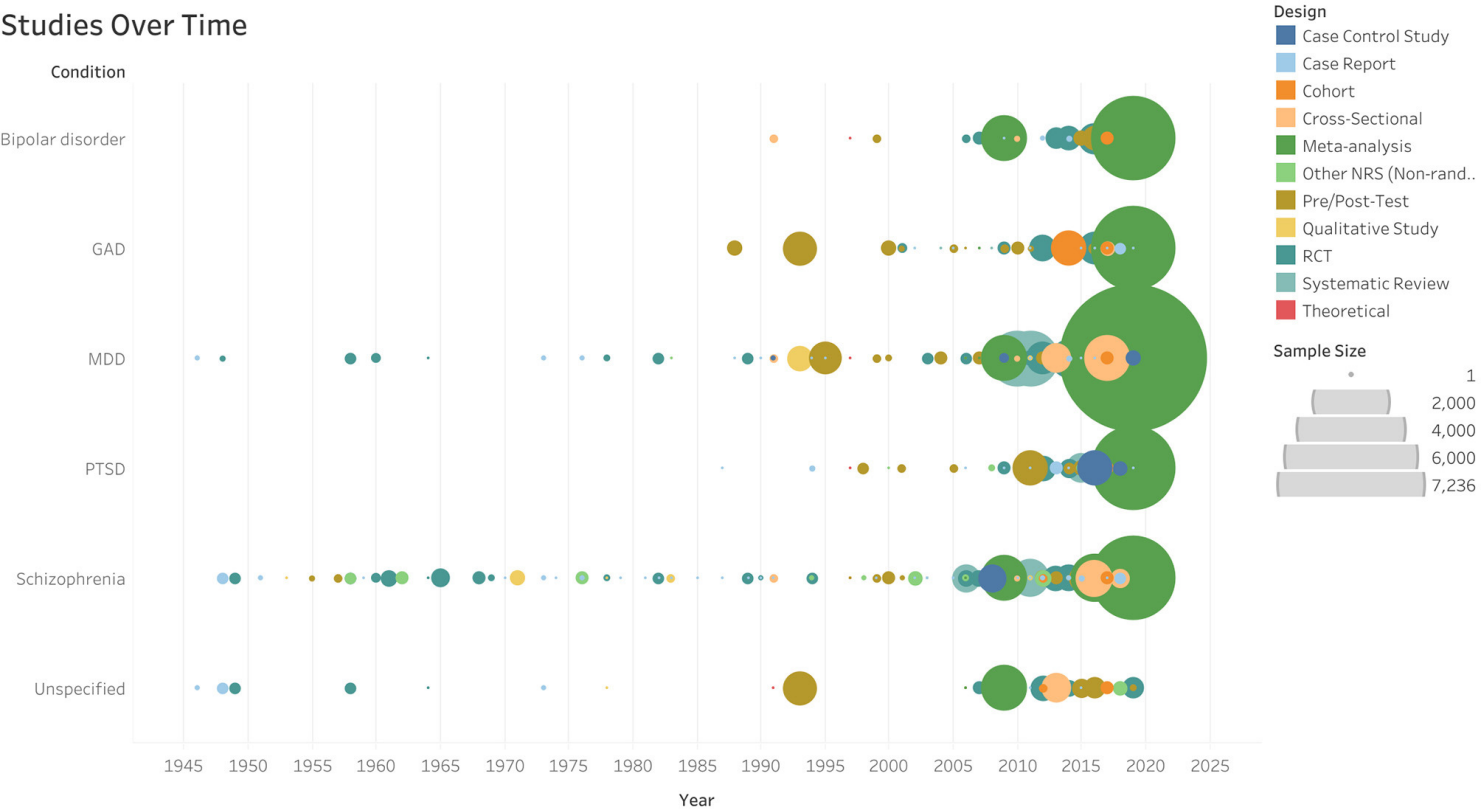
Studies Over Time.

Data extraction for this scoping review dated each study according to publication date. The earliest study identified in this literature search was published in 1946 (Rubin and Katz, 1946); studies began increasing in frequency in 2005, and this trend continues. Study frequency over time can be seen in Figure 1 (interactive version available at https://www.aerodatalab.org/livingreviews/onemind).

### Study Locations

Study locations were documented by country. When the study country could not be determined, locations were documented according to the corresponding author's country. Results are recorded in Table 2.

**Table 2:** Location.

**Location**	***N***	**%**	**Location**	***N***	**%**
USA	137	39.5	South Korea	3	0.9
UK	33	9.5	Sweden	3	0.9
Canada	16	4.6	France	3	0.9
Germany	16	4.6	Singapore	4	1.2
Taiwan	14	4.0	Iran	3	0.9
Australia	13	3.7	Austria	2	0.6
Norway	12	3.5	Hong Kong	2	0.6
China	8	2.3	Russia	1	0.3
Israel	8	2.3	Luxembourg	1	0.3
Various	8	2.3	Pakistan	1	0.3
Denmark	7	2.0	Argentina	1	0.3
Finland	7	2.0	Korea	1	0.3
Italy	5	1.4	Bosnia and Herzegovina	1	0.3
India	5	1.4	Mexico	1	0.3
Not Reported	5	1.4	Uganda	1	0.3
Japan	4	1.2	Zealand	1	0.3
Ireland	4	1.2	Nigeria	1	0.3
Turkey	4	1.2	Hungary	1	0.3
Greece	3	0.9	Thailand	1	0.3
Netherlands	3	0.9	Poland	1	0.3
South Africa	3	0.9	Portugal	1	0.3
			Total	349	100.0

In addition to geographic area, the settings of included studies varied widely. The majority (247, 85.4%) were conducted in clinical settings; other settings are documented in Table 3.

**Table 3:** Settings.

**Setting**	***N***	**%**
Community center	4	9.5
Homes	4	9.5
School (s)	4	9.5
nursing homes	4	9.5
Laboratory/Research	4	7.1
Online	3	4.8
Hospital library	2	4.8
VA	2	4.8
Music therapy clinic	2	4.8
Residential treatment	2	4.8
Not-reported	1	2.4
Dance studio	1	2.4
Arts center	1	2.4
Correctional facility	1	2.4
Halfway house	1	2.4
Activity room	1	2.4
Day center	1	2.4
Day care unit	1	2.4
Trauma center	1	2.4
Meditation studio	1	2.4
Drug rehabilitation	1	2.4
Total	42	100

### Study Designs

This scoping review collected data from 349 studies, inclusive of all study designs as well as secondary studies—including systematic reviews with or without meta-analyses (*n* = 60). When documenting study designs, the research team utilized the study design terminology identified by the study authors. If the study design was not identified by the authors, the research team assigned a design type based on their reading.

The study design for each study was coded according to the list in Table 4. Randomized Controlled Trials (RCTs) comprised the largest percentage of studies (23.2%), followed by Pre/Post Tests (21.8%) and Case Reports (18.3%).

**Table 4:** Design type.

**Design**	***N***	**%**
RCT	81	23.2
Pre/Post test	76	21.8
Case report	64	18.3
Systematic review	30	8.6
Other non-randomized controlled study	26	7.5
Qualitative study	24	6.9
Meta-analysis	15	4.3
Theoretical^a^	15	4.3
Cross sectional	9	2.6
Case control	6	1.7
Cohort	3	0.9
Total	349	100.0

a *“Theoretical” studies theorized the value of music for a given SMI, describing interventions without assessing outcomes (see Osborne, 2012; Zarate, 2016; Wang and Agius, 2018)*.

### SMIs Studied

Of the five SMIs included in this scoping review, schizophrenia was the most studied, followed by MDD and PTSD. The majority of included studies (69.2%) addressed only one SMI. Ninty studies (30.7%) addressed multiple diagnoses; notably, only one study focused strictly on bipolar disorder. Frequency counts by SMIs are documented in Table 5.

**Table 5:** Frequency counts by SMI.

**SMI**	**N of all studies**	**N of studies including only this dx**	**N of primary studies**	**N of primary studies including only this dx**
Schizophrenia	165	98	141	87
MDD	145	72	116	55
PTSD	145	55	112	43
GAD	59	17	50	14
Unspecified	34	0	30	0
Bipolar disorder	36	1	29	1

### Interventions

#### Music Activities

The music-based activities engaged by each study were documented verbatim for the first 210 studies. Researchers utilized this data to generate a standard list, with which activities in all 349 studies were ultimately coded. Activity types for all primary studies are documented in Table 6.

**Table 6:** Activity types.

**Activity**	**All studies *N***	**%**	**Primary studies *N***	**%**
Listening to music (Recorded)	89	15.3	82	17
Playing an instrument	68	11.7	57	11.8
Dancing/Movement to music	68	11.7	54	11.2
Listening to music (Unspecified)	68	11.7	53	11
Improvising	48	8.3	41	8.5
Not reported/specified	47	8.1	37	7.7
Singing in a group	37	6.4	29	6
Discussing or analyzing music	31	5.3	28	5.8
Songwriting	26	4.5	25	5.2
Listening to music (Live)	16	2.8	12	2.5
Singing	16	2.8	10	2.1
Drumming/Percussion	14	2.4	11	2.3
Other	13	2.2	13	2.7
Composing music	9	1.6	7	1.5
Recording music	7	1.2	7	1.5
Not applicable	7	1.2	2	0.4
Performing for an audience	6	1.0	6	1.2
Auditory stimuli	5	0.9	5	1
Singing alone	5	0.9	3	0.6

**Figure 2:**
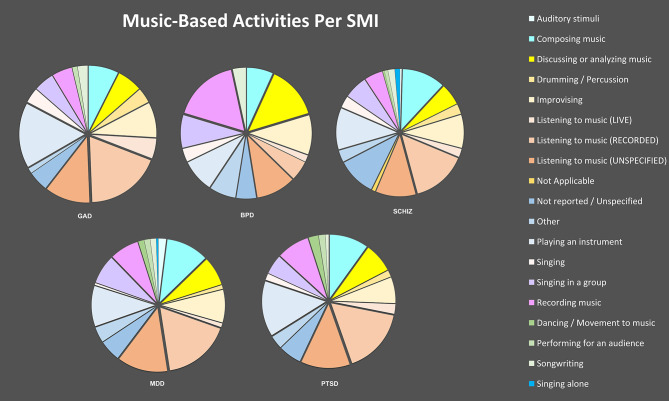
Music-Based Activities Per SMI.

Activities were later disaggregated by SMIs to illuminate whether condition/diagnosis appears to have had a bearing on choice of activity. Results are shown in Figure 2.

#### Activity Details

As noted earlier, each music activity was categorized as passive, active, or both. One hundred thirty-five primary studies (46.7%) involved active elements, while 79 (27.2%) involved passive elements; 52 studies (18%) involved both. The remaining 23 studies (8%) did not provide enough information to allow categorization.

**Figure 3:**
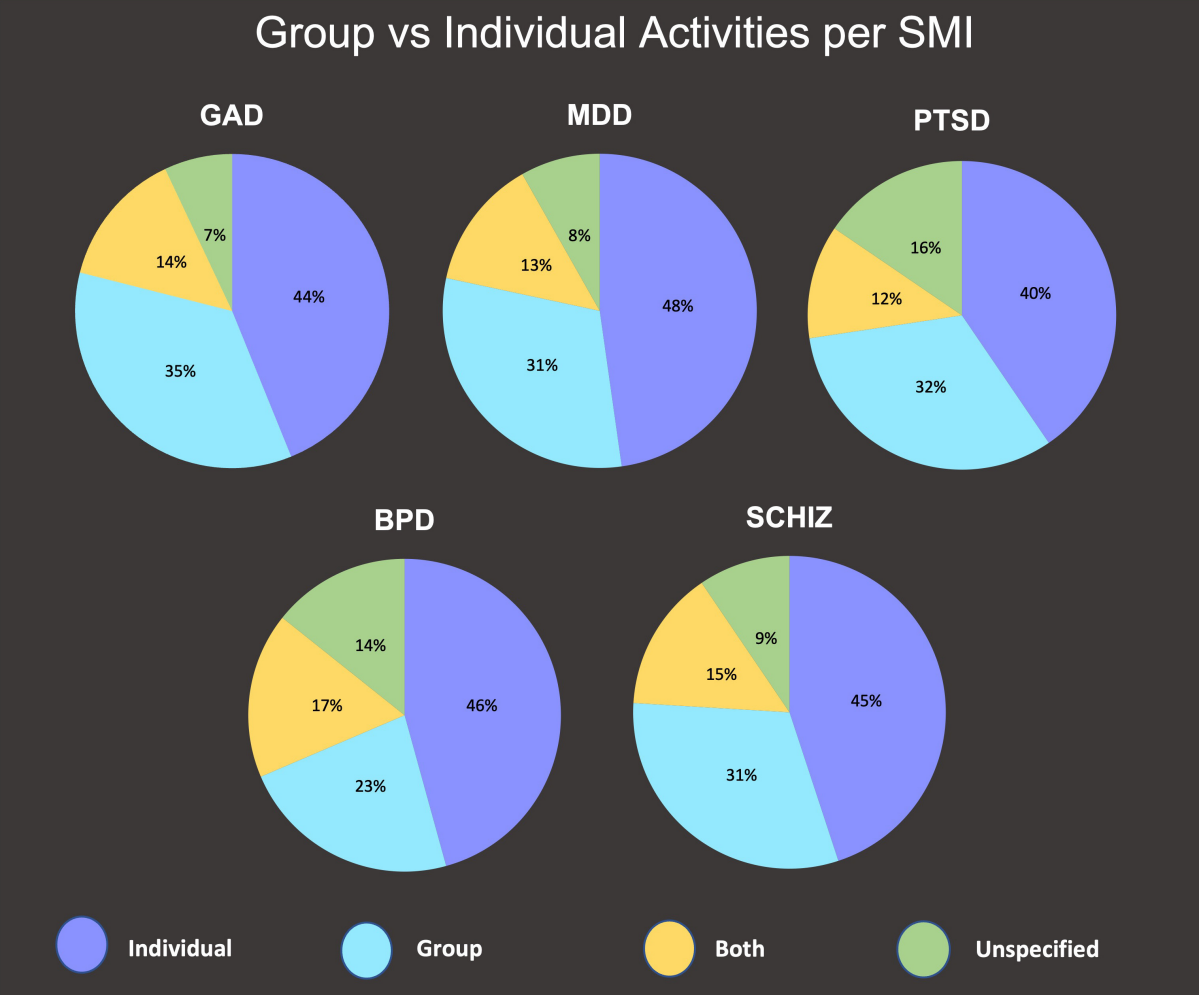
Group vs. Individual Activities Per SMI.

The music activities in each study were also categorized as individual, group, or both. One hundred and nine primary studies (37.7%) involved individual interventions; 139 (48.1%) involved group interventions and 21 (7.3%) involved both. Participation categories were disaggregated by SMIs to illuminate disparities among SMIs. Results are shown in Figure 3.

#### Facilitators

Two hundred and thirty-five primary studies (81.3%) reported information regarding the identities of those facilitating the interventions. Of these, Music Therapists working alone was the most represented identity or credential (29.4%), followed by Researcher/Investigator (10.4%). Remaining facilitator identities are documented in Table 7.

**Table 7:** Facilitators of music-based activities.

**Facilitator**	***N***	**%**
Music therapist	85	29.4
Not reported/Unspecified	47	16.3
Researcher/Investigator	30	10.4
Therapist	22	7.6
Researcher/Investigator, therapist	20	6.9
Other (Not listed here)	12	4.2
Dance/Movement therapist	11	3.8
Healthcare professional (Other than listed)	9	4.1
Music therapist + Psychologist	8	2.8
Psychiatrist	8	2.8
Not applicable	7	2.4
Self-administered (No facilitator)	6	2.1
Dance/Movement therapist + Psychologist	4	1.4
Music therapist + Psychiatrist + Healthcare professional (Other than listed)	4	1.4
Creative arts therapist	3	1.0
Music therapist + Other (Not listed here)	3	1.0
Dance instructor	2	0.7
Dance/Movement therapist + Music therapist	2	0.7
Music instructor	2	0.7
Music therapist + Healthcare professional (Other than listed)	2	0.7
Dance therapist + Psychiatrist + Healthcare professional (Other than listed)	1	0.4
Researcher/Investigator + Dance/Movement therapist	1	0.4
Total	289	100.0

### Outcomes

Outcomes in each of the included studies varied widely. To allow for analysis, outcomes were first documented verbatim, then later coded by researchers utilizing Dodd et al.' (2018) taxonomy of outcome domains. As expected given a focus on SMIs, the majority of studies (199, 57.0%) measured psychiatric outcomes—which “relate to mental health conditions and associated behaviors (e.g., addictions and behavioral problems)” (Dodd et al., 2018). For example, Zarate (2016) and Gutiérrez and Camarena (2015) examined whether music therapy reduced anxiety symptoms, and Pylvänäinen et al. (2015) investigated effects of dance movement therapy (DMT) on symptoms of depression. Other studies examined effects of music engagement on PTSD symptoms (e.g., Esparza, 2016; Macfarlane et al., 2019), symptoms of schizophrenia (e.g., He et al., 2018), stress levels (Wilbur et al., 2015; Steinberg-Oren et al., 2016), and similar.

Emotional Functioning/Well-Being was the second most prominent outcome domain (*n* = 130, 37.3%), addressing such factors as “impact of disease/condition on emotions or overall well-being (e.g., ability to cope, worry, frustration, confidence, perceptions regarding body image and appearance, psychological status, stigma, life satisfaction, meaning and purpose, positive affect, self-esteem, self-perception and self-efficacy” (Dodd et al., 2018). For example, Hovey (2013) and Silverman (2019) conducted studies of the effects of music therapy on self-efficacy among acute care mental health inpatients and patients with schizophrenia, respectively. Papadopoulos and Röhricht (2014) and Pylvänäinen and Lappalainen (2018) investigated effects of dance movement therapy on body image.

Social Functioning followed (*n* = 91, 26.1%); this domain encompasses ways in which one's condition affects “ability to socialize, behavior within society, communication, companionship, psychosocial development, aggression, recidivism, participation” (Dodd et al., 2018). For example, Solli and Rolvsjord (2015) studied whether making music in a group could improve participants' ability to take “an important first social step” (p. 76). Much earlier, Lehrer-Carle (1971) studied how group music therapy affected the ability of adolescents with schizophrenia to communicate with others and generate socially-valuable skills.

Physical functioning was the fourth most prominent domain (*n* = 30, 8.6%), with studies examining effects of music interventions on pain (e.g., Gosselin et al., 2019) and sleep (e.g., Guétin et al., 2009; Blanaru et al., 2012). Global Quality of Life followed (*n* = 35, 10.0%), often included as a second or third outcome. This domain “includes only implicit composite outcomes measuring global quality of life;” e.g., Zidani et al. (2017) utilized the Quality of Life Systematic Inquiry (QLSI) in their study of the effects of music listening on anxiety, and Deatrich et al. (2016) measured effects of group music therapy on the quality of life among inpatients at a psychiatric hospital. Role Functioning (*n* = 12, 3.4%) was also typically included as a second or third outcome, and involved such factors as ability to care for one's children (Tornek et al., 2003) or ability to follow therapy instructions (Strauss et al., 2016).

Cognitive Functioning outcomes (*n* = 22, 6.3%) involved such factors as cognition/memory testing (e.g., Glicksohn and Cohen, 2000; Tan et al., 2016) and attention/concentration (e.g., Shagan et al., 2018; Macfarlane et al., 2019). Delivery of Care^7^ was addressed by 12 studies (3.44%); e.g., Trimmer et al. (2018) examined whether integrating music with CBT group therapy increased effectiveness and retention. Preyde et al. (2017) investigated the feasibility and acceptability of group music therapy among youth in a hospital's Child and Adolescent Mental Health unit. All outcome domains identified by this scoping review are documented with frequency counts in Table 8.

**Table 8:** Count of outcome subdomains.

**Outcome subdomain**	**All Studies *N***	**%**	**Primary studies *N***	**%**
Psychiatric outcomes	199	57.0	157	54.3
Emotional functioning	130	37.2	113	39.1
Social functioning	91	26.1	72	24.9
Global quality of life	35	10.0	21	7.3
Physical functioning	30	8.6	26	9.0
Cognitive functioning	22	6.3	17	5.9
Delivery of care	12	3.4	11	3.8
Role functioning	12	3.4	12	4.2
None reported	11	3.2	8	2.8
Not applicable	7	2.0	3	1.0
Perceived health status	3	0.9	3	1.0
Need for further intervention	2	0.6	2	0.7
Adverse events/effects	1	0.3	0	0.0
Total	349	100.0	289	100.0

### Comparators

Like outcomes, the comparators in included studies varied widely; see Table 9. Note that while most study designs involve varying comparators, Pre/Post tests—which accounted for over a quarter of all study designs—always utilize the *same* comparator: a participant's status previous to the intervention. As a result, this “comparator” (documented as “Before/After”) was the most common across studies (*n* = 128, 44.3%). The second most prominent comparator was treatment as usual (TAU), utilized by 26 studies (9%).

**Table 9:** Count of comparator types.

**Comparator type**	**All studies *N***	**%**	**Primary studies *N***	**%**
Before/After	133	38.1	128	44.3
Not applicable	83	23.8	36	12.5
TAU	31	8.9	26	9.0
Without music	17	4.9	17	5.9
No treatment	12	3.4	11	3.8
Different kind of music	9	2.6	9	3.1
Different kind of music, Other (Not listed)	9	2.6	9	3.1
Delayed treatment	7	2.0	7	2.4
Other (Not listed)	7	2.0	7	2.4
Different art therapy (Drama, Vis art, Creative Play)	5	1.4	5	1.7
Group without SMI/Healthy volunteers	5	1.4	5	1.7
Passive listening	4	1.1	4	1.4
Silence	4	1.1	4	1.4
Different art therapy (Drama, Vis art, Creative Play) + Other CAM	3	0.9	2	0.7
Not recorded/Unspecified	3	0.9	2	0.7
Without music, Different kind of music	3	0.9	3	1.0
Group therapy	2	0.6	2	0.7
Other CAM	2	0.6	2	0.7
Self-administered vs. facilitator	2	0.6	2	0.7
Talk therapy	2	0.6	2	0.7
Cognitive therapy	1	0.3	1	0.3
Delayed treatment + Different kind of music	1	0.3	1	0.3
No treatment + Self-administered vs. facilitator	1	0.3	1	0.3
Other CAM + Delayed treatment	1	0.3	1	0.3
White/Pink noise	1	0.3	1	0.3
Without music + Other (Not listed)	1	0.3	1	0.3
Total	349	100.0	289	100.0

### Measures

Across the 289 included primary studies in this review, 271 distinct measures were reported (Data Sheet 2). Most studies utilized more than one measure, and reporting regarding their use varied considerably. To determine measures used, all measures were documented verbatim during data extraction. Then, to accommodate their scope and variety, verbatim measures were standardized^8^ and subsequently coded as Biomarkers, Custom Questionnaires, Physical/Performance Tests/Tasks, Qualitative Measures, or Standardized Questionnaires. These categories are documented in Table 10. All included measures are listed in Data Sheet 2.

**Table 10:** Measures/Instruments.

**Category**	**# of instruments in this category**	**%**	**# of times instrument in this category was used, across all primary studies**
Standardized questionnaires/Scales	201	74	378
Qualitative methods *(Interviews, focus groups, etc.)*	35	13	132
Custom questionnaires/Scales	13	5	27
Biomarkers	11	4	32
Physical/Performance tests/tasks	11	4	12
Total	271	100%	581

### Study Findings

Due to the wide variety of outcomes and measures included, findings for each primary study were coded simplistically according to whether the music intervention had performed better, worse, or equal to the comparator, or whether results had been indeterminable (Table 11).

**Table 11:** Study results.

**Results**	***N***	**%**
Music > Comparator	198	68.51
Not Applicable	39	13.49
Undetermined	35	12.11
Music = Comparator	9	3.11
Music < Comparator	8	2.77
Total	289	100.00

## Discussion

This scoping review found that hundreds of studies related to music-based interventions for SMIs have been conducted worldwide over several decades, with significant increases in study frequency since 2005. Studies have primarily involved mixed-gender groups with more than one race or ethnicity represented; however, demographic reporting was inconsistent and routinely inadequate. This limits researchers' and practitioners' ability to note disparities in results, and to ascertain who may be most helped by music-based activities and experiences.

As noted, schizophrenia was the most studied SMI, followed by MDD, PTSD, GAD, and bipolar disorder. While bipolar disorder was included in 30 studies, only one study focused singularly on this illness; similarly, only 14 studies addressed GAD alone^9^. Further research is warranted to determine causes for frequency disparities among the included SMIs; e.g., do theories of change indicate less potential for music-based interventions for bipolar disorder and GAD patients, or have studies regarding these illnesses simply been limited by lack of funding, researcher interest, and other concerns?

Many systematic reviews related to music and health have been unable to aggregate and synthesize evidence from studies; as a result, they have often recommended further study and greater rigor (see van Westrhenen and Fritz, 2014; Petrovsky et al., 2015; Williams et al., 2018). This need for rigor is often interpreted as a need for greater utilization of RCTs; however, this scoping review yields that RCTs emerged as the predominant study design. In other words, a lack of rigor—along with other obstacles to evidence synthesis—should not be simplistically linked to a failure to utilize a particular study design. Indeed, while some systematic review authors do highlight the value of randomized controlled trials (Yang et al., 2019), others note that rigor would be improved by longer-term studies (Veerman et al., 2017), as well as improved use of rigorous qualitative strategies such as data triangulation, structured clinical observations, and far more detailed descriptions of study participants, their behaviors, and musical activities (van Westrhenen and Fritz, 2014).

Of course, although RCTs were the predominant study design in this scoping review, it should be noted that controls varied widely—including treatment as usual (TAU), delayed treatment, other genres of music, other forms of art therapy, and more. As a result, the findings of the included RCTs had quite disparate applications. In addition, given that scoping reviews are not designed to assess data quality, the authors cannot speak to the rigor and quality of the studies included, regardless of design. In the absence of quality assessments, it can be tempting to use study design as a proxy for study quality (e.g., assuming that an RCT is of higher evidential quality than a case study); however, such assumptions are inadvisable, as quality varies widely across study designs (see Ryan et al., 2013; Chambers, 2017; Higgins and Thomas, 2020).

### Activities

A wide range of activities were represented in this scoping review, with the most prominent being listening to recorded music (15.3%). Notably, 47 studies (8.1%) did not report the type of musical activity experienced by study participants. Active engagement in music-based interventions was considerably more common in the review's findings than passive engagement (46.7 vs. 27.2%), and group interventions were more common than individual (48.1 vs. 37.7%). Notably, the SMI being addressed appears to have affected activity facets. For example, bipolar disorder studies involved more “recording music” activities than other SMIs, while GAD and PTSD studies involved more playing of instruments. Group interventions were utilized for MDD more often than for PTSD (48 vs. 40%).

Just as the details of activities themselves were inconsistently reported, the rationale behind detail selection is inconsistently provided. As a result, it is often unclear to what extent activity facets—such as passive vs. active engagement, group-based vs. individual approaches, genres, etc.—were selected based upon factors such as availability, convenience, or facilitator expertise, vs. factors such as prior research or formalized theories of change related to particular participant groups or conditions. Considering that outcomes are highly likely to be affected by activity details, further research is needed to determine which types of engagement reliably generate particular results—and for whom. This would allow selection of activity details based on diagnoses, symptoms, participant factors, outcomes, and more. To this end, it is critical that future researchers document the rationale behind each decision related to interventions and their many facets (see Recommendation I).

### Facilitators

Facilitators ranged in discipline and background; while music therapists comprised the largest percentage (29.4%), those identified simply as a researcher/investigator (often including an author of the study) made up over 16% of the facilitators. The frequency with which researchers facilitated music interventions raises two questions: First, did researchers receive musical (or music-facilitation) training before initiating programs or studies; and second, was their training decision based on science/evidence? (For example if researchers did *not* receive training, was this based on evidence that such training was not essential/beneficial?).

One in six primary studies (16.6%) involved multidisciplinary teams as facilitators, or facilitators who had multidisciplinary backgrounds—underscoring the interdisciplinary nature of the music and SMI intersection. Unfortunately, almost as many (16.3%) failed to report or specify facilitator details at all. This prevents assessment regarding the role of facilitators—including their discipline, training, relationship with study participants, *etc*.—in an intervention and its outcomes. Inconsistencies in reporting, combined with the variety of facilitators involved, highlight the need for continued research regarding whether and to what extent facilitator characteristics and backgrounds affect results. In the future, evidence synthesis will be supported by the careful reporting of facilitator details including credentials, training (or lack thereof), and relationships with study participants (see Recommendation I).

### Reported Findings

Over two-thirds of all primary studies (68.5%) reported positive findings (“music > comp”), while 12 percent (*n* = 35) produced indeterminate results. Only eight studies (2.8%) indicated that the music-based intervention had produced worse results than the comparator. A scoping review does not offer assessments of study quality or risk of bias; as a result, practice and policy guidelines cannot be generated based on existing data. However, these data indicate the potential promise of music-based activities in treatment or symptom management of SMIs, and they underscore the value of continued research—with the implementation of robust study and reporting protocols.

### Evidence Synthesis

Overall, the findings in this scoping review suggest that inconsistent reporting practices, a lack of core outcomes or outcome measures, and the sheer complexity of interventions combine to render difficult the synthesis of existing evidence for the purposes of further developing or scaling music-based interventions. Reporting practices and core outcome sets are discussed under Recommendation IV, below. Regarding intervention complexity, the many variables involved in each musical activity generate the potential for effects on studied outcomes. For example, “listening to music,” though often documented as a straightforward activity, can take place while participants lay down or while they move about; it can also take place via the use of headphones, small speakers, large speakers, or the presence of a live musician. The music to which participants listen may be selected by study participants themselves, or by facilitators; it could be of high quality and played at an ideal volume; it could also be of low quality, or too quiet/loud for a given participant. Activity complexity expands significantly when one adds the potential effects of: setting (e.g., clinic vs. home vs. community center vs. prison); facilitators' relationships with participants; group interactions (including how well group members know one another); how long a given participant has lived with an SMI; how many other treatments or options a study participant has previously tried; levels of autonomy in the activity (who selects music, volume, instruments, dose/duration, etc.); and more.

Unfortunately, this scoping review found that such variables were insufficiently and inconsistently reported—precluding readers' ability to discern precisely what type of activity/component delivered (or failed to deliver) results. For example, it was impossible in some cases to ascertain the identity of the activity facilitator at all, let alone the nature of the facilitator's relationship with study participants. Similarly, basic demographic data were inconsistently available, let alone details regarding participants' diagnoses (were these volunteered/self-reported?) or participants' prior experience(s) with mental health treatments.

Meticulous documentation is necessary for identifying mechanisms, causal patterns, and replicable strategies. Replicability is critical to the development of robust evidence bases and practice guidelines for integrating music into intervention and treatment plans. When researchers or health practitioners read a peer-reviewed article and cannot identify precisely what was done in the reported study or intervention, it becomes impossible for them to replicate the process with other populations, in other settings, or with larger sample sizes.

Of course, studies that cannot produce generalizable results (due to study designs or small sample sizes) may yet generate valuable, transferable findings; however, a lack of detail impedes this benefit as well. In other words, although scalability is not an aim of every music-based intervention for SMIs, improved reporting is nevertheless critical for enhancing understanding of the music-SMI intersection across disciplines. Consistent, rigorous detail in reporting will support the sharing of information among mental health practitioners, individuals with lived experience with SMIs, artists and arts-program facilitators, and others, while also improving research and scalability when appropriate.

Music's complex, wide-ranging nature—as observed in this review—should be understood as a promising asset; it suggests that music-based strategies offer adaptability, flexibility, and customize-ability as SMI interventions. Nevertheless, the sheer breadth of activities falling under the heading of “music”—and the extent to which each of these can vary from setting to setting—present impediments to synthesizing evidence and advancing the field. To navigate these difficulties, five recommendations are offered below.

### Recommendations

While existing systematic reviews have demonstrated the potential for music interventions to support mental health (e.g., music therapy for depression; Aalbers et al., 2017), many have also asserted that there are issues with the quality of evidence and relevance of outcome measures (Aalbers et al., 2017; Geretsegger et al., 2017; Clift, 2020). Results of the current scoping review comport with these assertions. As noted, scoping reviews are not designed to analyze existing research for the purpose of making practice recommendations; however, they *are* designed to document the state of the science and identify gaps and future directions for research. The below recommendations are intended to support ongoing and future research to improve the evidence base and promote translation of evidence into practice.

#### Recommendation I: Standardize Reporting Practices by Adopting Existing Reporting Guidelines to Clearly and Consistently Document Studies

A limitation to the current body of research—and a hindrance to translating existing evidence into practice—is a lack of consistent and sufficient documentation with regard to key study information necessary to understand: (1) the populations and conditions studied; (2) specific details of each intervention studied; and (3) the specificity and relevance of outcomes measured, along with details required for an assessment of the quality of the research.

Underreporting of key information in research is not a phenomenon unique to music-based studies. An analysis of completeness of the intervention description in trials and reviews found that studies of non-pharmacological interventions provided adequate documentation of the intervention only 29% of the time, as compared to 67% for pharmacological interventions (Glasziou et al., 2008). In addition, a comprehensive study of adherence to reporting guidelines over a 20-years span (January 1996 to September 2016) for clinical trials, systematic reviews, observational studies, meta-analysis, diagnostic accuracy, economic evaluations, and preclinical animal studies demonstrated that 87.9% of the included studies reported suboptimal adherence to reporting guidelines (Jin et al., 2018). Inadequate reporting of intervention studies limits opportunities for clinicians and patients to utilize research studies to support medical decision making and precludes future researchers from building on existing research. By contrast, standardizing reporting practices for all studies—including those involving music-based interventions for SMIs—will support translation into policy and practice and improve future research.

Sharp et al. (2019) observed that across disciplines, “[r]eporting guideline endorsement rates are low; information is vague and scattered” (n.p); similarly, Jin et al. (2018) confirmed “the need for further emphasis in the scientific community to encourage the use of reporting guidelines” (n.p.). Building on their findings, we recommend that future research incorporate and promote the use of reporting guidelines. Reporting guidelines inform researchers which elements of a study must be documented in order to support the transparency, interpretation, synthesis, replication, and reproducibility of research. A number of reporting guidelines are available to researchers working at the nexus of music and mental health, and they help document each key aspect of a study, including:

- Conditions/populations of interest; e.g., PTSD (Kaloupek et al., 2010);
- Study designs employed; e.g., CONSORT for RCTs, STROBE for observational studies;
- Types of data collected; e.g., eMOOD (Faurholt-Jepsen et al., 2019), fMRI (Poldrack et al., 2008); and
- Intervention details; e.g., TIDieR (Hoffmann et al., 2014) and CReDECI 2 (Möhler et al., 2015).

Notably, while reporting guidelines focus on optimizing the translation of research findings into practice, much of the valuable work regarding art's impacts on health is not necessarily occurring in the context of formal academic research. Therefore, the authors suggest that artists, practitioners, and facilitators also utilize these reporting guidelines when evaluating and documenting arts-based programming and activities—regardless of the level of researcher involvement. This enables consistency in communicating the work across the spectrum of disciplines and audiences.

#### Recommendation II: Utilize the Current Scoping Review as a Basis on Which to Investigate the Utility of a Reporting Guideline or Reporting Guideline Extension for Music Interventions

Studies of music interventions in mental health may be sufficiently unique as to warrant a new guideline for reporting these studies. Indeed, a 2017 workshop about “music and the brain,” hosted by the National Institutes of Health (NIH), generated recommendations to promote “more rigorous reporting of interventions, methodologies and results” at the intersection of music and brain health, and to “establish standardized and/or personalized outcome measures” (Ambler et al., 2020). A proposal for a reporting guideline for music and health was suggested by Robb and colleagues (2011); however, this guideline does not appear to have been formalized or taken up by the field.

A first step toward the development of any reporting guideline is an evidence synthesis of the body of evidence in the relevant field, such as the current scoping review. The data in this review, combined with existing guidelines, suggests an opportunity to generate a new tool for researchers working at the nexus of music and mental health. If taken up by researchers in the field, a reporting guideline specific to music in mental health research could facilitate better documentation—ultimately improving evidence synthesis and the translation of research into practice. Such a guideline could also support the design of new music-based interventions, and support funding agencies in structuring their grant reporting and calls for proposals (CFPs).

If undertaken, the development of a new reporting guideline should take a comprehensive, stakeholder-engaged approach that yields insights into how clinicians, artists, content experts, patients, caregivers, researchers, and other key stakeholders imagine the research process. Diverse perspectives and iterative development processes help ensure the guideline accounts for the many ways in which music impacts on mental health. The guideline should additionally support transparent documentation for all who will be looking to evidence to inform their decision making.

Meanwhile, the authors of the current scoping review have identified key elements for reporting, which may be considered in any guideline for music interventions in mental health research. These are offered in Data Sheets 3 and 4.

#### Recommendation III: Accommodate the Complexity of Music Interventions

*Simple interventions* typically feature a linear pathway whereby outcomes and interventions are linked; by contrast, *complex interventions* include non-linear causal pathways and multiple, interacting components (Petticrew, 2011). In fact, even “simple” interventions are often influenced by complex interplays of individual characteristics, social determinants, the health care delivery system, and the interventions themselves (Guise et al., 2017). Like most non-pharmacological interventions, music interventions are complex, consisting of several components that work independently or interdependently of each other and can be tailored to fit a specific setting (Craig et al., 2008). This non-standardization of music interventions complicates how we study them.

For example, the fact that music could be delivered through headphones to patients in a therapist's private office, vs. delivered through speakers to patients in clinical waiting rooms, generates so-called *intervention complexity* (Guise et al., 2017). In addition, music has unique neurobiological and psychological impacts (Boso et al., 2006) that can interact with other clinically important outcomes in healthcare—generating so-called *pathway complexity* (Guise et al., 2017)^10^. In short, music-based approaches to SMI care must be understood and studied as complex interventions.

Working with complex interventions requires solid theoretical understanding of how a given intervention causes change. Theories of change undergird the development of intervention protocols; they also allow weak links in the causal chain to be identified and strengthened. Of course, researchers should balance the need for protocol fidelity with the utility of adaptation when important for the local setting; however, departures from protocols should be documented with care and transparency (see “Modifications,” Data Sheet 4). In addition, when studying complex interventions, researchers should be cautious about interpreting findings—as a lack of impact may not signal a lack of efficacy, but rather a failure of implementation. Lastly, research teams working with music and SMIs should consider study designs capable of yielding better evidence for complex interventions; e.g., cluster-randomized trials, N-of-1 designs, and step-wedge designs (Craig et al., 2008).

As stated, transparent reporting of study elements is critical; to that end, the Criteria for Reporting the Development and Evaluation of Complex Interventions in Healthcare: Revised Guideline (CReDECI 2) provides specific guidance on documenting the components of complex interventions (Möhler et al., 2015). It emphasizes the importance of documenting:

- Theoretical underpinnings of an intervention.
- Selection of outcomes and modes of evaluation.
- All intervention components, including reasons for their selection, their essential functions, and any intended interaction between components.
- Strategy for delivering the intervention within the study context.
- All materials and tools used in the delivery of the intervention.
- Fidelity of the delivery process compared to the study protocol.

#### Recommendation IV: Consider Developing Core Outcome Sets and Core Measures for Studies of Music's Use in Supporting Mental Health

This scoping review demonstrates broad uptake of music as an intervention to support those with SMI and their caregivers across a continuum of outcomes—including clinical, humanistic, and economic outcomes that matter to patients, providers, and policy-makers. Nevertheless, it remains a rare occurrence for systematic reviews of music-based interventions to yield a strong recommendation for clinical translation into practice. As stated above, this is not necessarily a result of insufficiently rigorous study methods (e.g., RCTs), but rather of clinical heterogeneity—defined as a “…variation in study population characteristics, coexisting conditions, cointerventions, and outcomes evaluated across studies included in an SR [systematic review] or CER [comparative effectiveness research] that may influence or modify the magnitude of the intervention measure of effect” (West et al., 2010). In other words, the selection of unique, often incomparable, heterogenous outcomes—in terms of outcome *domains* (what is measured) and selection of *measurement tools/instruments* (how the outcome is measured)—often limits the combination and comparison of results of individual studies. An additional consequence of inconsistent outcome selection is the risk that outcomes are being selected without having determined their importance or relevance to patients and caregivers living with the studied conditions.

This issue of outcome heterogeneity is not specific to research related to music and mental health; it actually affects the conclusion of a substantial proportion of Cochrane Collaborative systematic reviews (Kirkham et al., 2010), and outcome reporting bias is an important problem in randomized trials (Dwan et al., 2008). Of relevance to the current scoping review, these issues also occur in a great deal of general SMI research. A meta-analysis of 198 trials of psychotherapeutic interventions for MDD identified 33 unique outcome measurement tools (Barth et al., 2013). The current scoping review confirms this trend with regard to music and SMIs, demonstrating extensive heterogeneity in outcomes and outcome measures (see Tables 8, 10).

One approach to mitigating the effects of outcome heterogeneity is the adoption of *core outcome sets*. A core outcome set (COS) establishes a minimum standardized collection of outcomes, identified by key stakeholders through robust consensus methods^11^. Utilizing a minimum COS would improve comparative effectiveness of interventions and ensure that the outcomes that are important to communities requiring evidence for decision-making (e.g., patients, caregivers) are represented in the study design process, even if not directly engaged by the study team.

Implementing a COS does not restrict researchers to *only* those minimum outcomes; rather, it sets an expectation that COS will always be collected while researchers continue to explore additional outcomes. If it is decided that a measure within a COS should be excluded from a given study, the research team is expected to explain the exclusion, as well as the relevance and importance of other (additional) selected outcomes (Williamson et al., 2012).

Notably, the process of engaging stakeholders to identify a COS can itself yield important findings that advance health. For example, a recent study to identify outcome domains and outcome measures of importance to people living with MDD identified 80 outcome domains related to the benefits of treatments, derived from the lived experience of depression (Chevance et al., 2020). The study revealed that several outcome domains frequently mentioned by participants were not measured by the seven most-used depression scales in MDD research. It also identified 57 additional outcome domains that were unrelated to the direct benefits of treatment (e.g., treatment safety, health-care organization, social representation of depression), and which at present remain understudied (Chevance et al., 2020).

For the above reasons, the authors recommend the uptake and implementation of existing COS for each SMI. Teams studying music-based interventions for SMIs should first identify whether a COS exists for the population and condition of interest, and (if so) implement that COS by reviewing the literature and consulting the COMET Database. In addition, the development and implementation of a specific COS for the study of music-based interventions for SMIs may be an important step in this field, to promote the translation of evidence into practice.

#### Recommendation V: Consider Developing a Taxonomy to Better Organize and Define “Music” and “Music Engagement”

Distinct activities included under the general umbrella-term of “music” are likely to affect health via multiple, distinct mechanisms. For example, vigorous group drumming outdoors can be expected to generate different physiological and psychological effects than listening to music on headphones while lying in a hospital bed. While both are music-based activities, the expectation that they share outcomes or measures may strain the definition or category of music beyond its utility in health research and practice. To be clear, improved reporting of *all* music-based interventions is both urgent and fully feasible, as is the increased standardization of many research aspects and processes. However, expecting distinct activities to utilize similar outcomes or measures merely because they can both be described as “music(al)” may actually *minimize* the effects of music-inclusive interventions by obscuring or eliding their scope and variety.

The general category of “music” is highly useful in terms of developing research and practice networks, supporting advocacy, raising funds, and advancing patient and public awareness of the value of music as an aspect of health and well-being. However, grouping disparate activities together in research, particularly apart from specific protocols, may perpetuate the heterogeneity that has precluded adequate synthesis to date. Notably, this scoping review was able to identify the difficulties of this heterogeneity in part because it too was broadly inclusive. To meet its goal of illuminating all uses of music in the treatment or mitigation of symptoms related to SMIs, this review was required to include studies involving myriad activities that, while related to music, are likely to effect change in disparate ways.

As researchers and practitioners consider developing formal reporting guidelines and core outcome sets, they might also consider developing taxonomies of music-based or music-inclusive activities—perhaps grouping them iteratively (e.g., by types of movement involved, potential mechanisms, where they range from prescriptive to improvisational/expressive, etc.). Such taxonomies would help generate shared vocabularies for use by practitioners, artists, and the research community, and support development of best practices related to specific forms of music engagement. Taxonomies may be operationalized by journals and research databases to index studies—enabling better identification of similar studies, in turn supporting meaningful aggregation and synthesis of studies for the purposes of systematic review.

### Limitations

The extraction and coding of data across this collection of studies involved adapting studies to the extraction tool, typically in collaboration with multiple research team members. It is possible that, provided with the same data, other research teams may have made alternative selections. However, these would be highly unlikely to result in changes to the review's overarching findings or recommendations.

Interruptions to library access caused by the COVID-19 pandemic limited the research team's access to studies that may otherwise have been available. While many of these would likely have been excluded regardless, it is possible that current findings exclude data from applicable studies. That said, the sample size of the review generates a high level of confidence regarding its findings; again, it is unlikely that the addition of inaccessible texts would result in changes to overarching findings or recommendations.

Finally, the large number of studies included in this scoping review resulted in a focus on frequency counts and standardized coding practices, with the aim of illuminating a general landscape and identifying research densities and gaps. Future researchers may wish to disaggregate studies by various factors, allowing more in-depth looks into particular practices, populations, or study designs.

## Conclusion

This scoping review sought to support the improvement and expansion of options for addressing SMIs by gathering and coordinating evidence regarding music-based interventions for MDD, PTSD, bipolar disorder, GAD, and schizophrenia. To our knowledge, it is the first scoping review to examine intersections of music and SMIs at this scale. Having documented details regarding 349 studies, this review illuminates the volume and variety of research occurring at intersections of music and SMIs. It additionally underlines the promise of music in supporting mental health, as most studies indicated positive results. Findings indicate that music-based interventions are currently constrained not by a lack of research, but by an ongoing inability to synthesize the extensive research being generated. Despite accumulating studies since 1946, synthesis continues to be precluded by a wide variety of outcomes and outcome measures, a lack of quality reporting, and the inherent complexity of music-based interventions. Findings suggest that the extensive time, funds, and expertise being invested in this field will continue to see limited returns until widespread changes occur to reduce heterogeneity (as feasible), increase replicability and transferability, and improve understandings of mechanisms and causal pathways.

To support these efforts, this review offered five key recommendations, with an emphasis on the immediate action step of improving reporting practices. While the review's illumination of extensive heterogeneity and complexity have indicated urgent obstacles, the creativity, innovation, and attentiveness to patient experience that are apparent in included studies also figure as assets to the mental health field's overarching efforts to advance care, access, and equity over the long term.

## Data Availability Statement

The original contributions presented in the study are included in the article/Supplementary Material, further inquiries can be directed to the corresponding author/s.

## Author Contributions

TG led the scoping review study as well as the writing of this article. SS co-authored the discussion and recommendations, and contributed multiple revisions throughout. HK, SG, AT, and CS contributed data analyses. SG contributed to the manuscript's introduction. SM helped conceive of the project and oversees IAM Lab's research output. All authors contributed to the article and approved the submitted version.

## Conflict of Interest

The authors declare that the research was conducted in the absence of any commercial or financial relationships that could be construed as a potential conflict of interest.

## Footnotes

^1^See also Vigo et al. (2019), who indicated that a “striking imbalance exists between government spending on mental health and the related disease burden in the Americas” (n.p.).

^2^For example, by reducing stigma associated with care, or by rendering care less intimidating or more culturally responsive.

^3^This scoping review was part of a larger initiative which included a transdisciplinary gathering of thought leaders, who were asked to review and respond to early scoping-review findings. This group then generated recommendations to advance opportunities at the intersection of music and SMIs. Their responses, informed by professional expertise as well as lived experience with SMIs, will be published separately.

^4^This scoping review excluded (a) studies that were inexplicit regarding the *type* of mental illness treated (such as the five diagnoses here named); and (b) studies that examined effects of relevant interventions on one or more of the five diagnoses here listed, but included these among *other* illnesses or diagnoses while failing to disaggregate outcomes.

^5^The authors acknowledge that the inclusion of dance will be seen by some disciplines as problematic; however, many cultures, disciplines, patients, and practitioners do not formally separate these activities (Besson and Schön, 2001; Thompson, 2014; Leman and Maes, 2015; Levitin et al., 2018). Because dance interventions utilize music (with rare exceptions), excluding dance would have resulted in the exclusion of many interventions in which music played a role—thereby compromising the scoping review's goal of generating of a broad, inclusive landscape of all uses of music in SMI interventions.

^6^For example, if a study indicated that study participants had been veterans *and* clinical patients, the “Veterans and/or Military Personnel” category would be selected over “patients,” due to increased specificity.

^7^According to Dodd et al. (2018), this outcome domain refers to outcomes related to “adherence/compliance; patient preference; tolerability/acceptability of intervention; withdrawal from intervention (e.g., time to treatment failure, reason for stopping therapy); appropriateness of intervention; accessibility, quality and adequacy of intervention; patient/career satisfaction (emotional rather than financial burden); process, implementation, and service outcomes (e.g., overall health system performance and the impact of service provision on the users of services)”.

^8^For example, “heart rate” and “pulse” were combined, “EEG” and “electroencephalogram” were combined, etc.

^9^By comparison, 43 studies addressed PTSD alone, 55 addressed MDD alone, and 87 addressed schizophrenia alone.

^10^Recognizing that complex interventions require deeper consideration and different tools to design, implement, replicate, and critically review associated studies, the Medical Research Council updated their guidance in 2019. Their document articulates key implications for the study of complex interventions, and provides recommendations for researchers studying complex interventions (Craig, 2019).

^11^These consensus methods should themselves be measured and consistently reported in all trials for a specific clinical area (Williamson et al., 2012).
