# Supplementary material for: The Use of Music in the Treatment and Management of Serious Mental Illness: A Global Scoping Review of the Literature
Source: Front Psychol. 2021 Mar 31;12:649840. doi: 10.3389/fpsyg.2021.649840 (PMC8044514; doi:10.3389/fpsyg.2021.649840)
Supplement: Supplementary file 1 [file Data_Sheet_1.docx]

**Data Sheet 1: Complete List of Included Studies**

Aalbers, S., Fusar-Poli, L., Freeman, R. E., Spreen, M., Ket, J. C., Vink, A. C., … Gold, C. (2017). Music therapy for depression. *Cochrane Database Syst Rev*, *11*(PG-Cd004517), Cd004517. https://doi.org/10.1002/14651858.CD004517.pub3

Ager, A., Akesson, B., Stark, L., Flouri, E., Okot, B., McCollister, F., & Boothby, N. (2011). The impact of the school-based Psychosocial Structured Activities (PSSA) program on conflict-affected children in northern Uganda. *Journal of Child Psychology and Psychiatry*, *52*(11), 1124–1133. https://doi.org/10.1111/j.1469-7610.2011.02407.x

Ahonen, H., & Desideri, A. M. (2014). Heroines’ Journey--Emerging Story by Refugee Women during Group Analytic Music Therapy. *Voices: A World Forum for Music Therapy*, *14*(1 PG-np), np. Retrieved from https://search.proquest.com/docview/1514320005?accountid=11752 http://findit.library.jhu.edu/resolve?url_ver=Z39.88-2004&rft_val_fmt=info:ofi/fmt:kev:mtx:journal&genre=article&sid=ProQ:ProQ%3Aiimpft&atitle=Heroines%27+Journey--Emerging+Story+by+Refugee+Wo

Aigen, K. (1990). Echoes of silence. *Music Therapy*, *9*(1 PG-44–61), 44–61. https://doi.org/10.1093/mt/9.1.44

Altschuler, I. (2001). A psychiatrist’s experience with music as a therapeutic agent. *Nordic Journal of Music Therapy*, *10*(1 PG-69–76), 69–76. https://doi.org/10.1080/08098130109478019

Anderson, A. N., Kennedy, H., DeWitt, P., Anderson, E., & Wamboldt, M. Z. (2014). Dance/movement therapy impacts mood states of adolescents in a psychiatric hospital. *Arts in Psychotherapy*, *41*(3 PG-257–262), 257–262. https://doi.org/10.1016/j.aip.2014.04.002

Apóstolo, J., Queirós, P., Rodrigues, M. A., Castro, I., & Cardoso, D. (2012). The effectiveness of nonpharmacological nursing interventions in elderly with depressive disorders: a systematic review. *JBI Library of Systematic Reviews*, *10*(28 PG-1–10), 1–10. https://doi.org/10.11124/jbisrir-2012-297

Archambault, K., Vaugon, K., Deumie, V., Brault, M., Perez, R. M., Peyrin, J., … Garel, P. (2019). MAP: A Personalized Receptive Music Therapy Intervention to Improve the Affective Well-being of Youths Hospitalized in a Mental Health Unit. *J Music Ther*, *56*(4 PG-381–402), 381–402. https://doi.org/10.1093/jmt/thz013

Ashida, S. (2000). The effect of reminiscence music therapy sessions on changes in depressive symptoms in elderly persons with dementia. *J Music Ther*, *37*(3 PG-170–82), 170–182. https://doi.org/10.1093/jmt/37.3.170

Atiwannapat, P., Thaipisuttikul, P., Poopityastaporn, P., & Katekaew, W. (2016). Active versus receptive group music therapy for major depressive disorder-A pilot study. *Complement Ther Med*, *26*(PG-141-5), 141–145. https://doi.org/10.1016/j.ctim.2016.03.015

Barcelona de Mendoza, V., Harville, E., Savage, J., & Giarratano, G. (2016). Association of Complementary and Alternative Therapies With Mental Health Outcomes in Pregnant Women Living in a Postdisaster Recovery Environment. *J Holist Nurs*, *34*(3 PG-259–70), 259–270. https://doi.org/10.1177/0898010115609250

Barde, S., Upendra, S., Sawane, K., Waghmare, S., & Devi, S. (2019). Effectiveness of music therapy on anxiety among elderly residing at selected geriatric homes at Pune City. *Indian Journal of Public Health Research and Development*, *10*(7 PG-1621–1623), 1621–1623. https://doi.org/10.5958/0976-5506.2019.01829.1

Beck, B. D., Lund, S. T., Sogaard, U., Simonsen, E., Tellier, T. C., Cordtz, T. O., … Moe, T. (2018). Music therapy versus treatment as usual for refugees diagnosed with posttraumatic stress disorder (PTSD): study protocol for a randomized controlled trial. *Trials*, *19*(1 PG-301), 301. https://doi.org/10.1186/s13063-018-2662-z

Beck, B. D., Messel, C., Meyer, S. L., Cordtz, T. O., Søgaard, U., Simonsen, E., & Moe, T. (2018). Feasibility of trauma-focused Guided Imagery and Music with adult refugees diagnosed with PTSD: A pilot study. *Nordic Journal of Music Therapy*, *27*(1 PG-67–86), 67–86. https://doi.org/10.1080/08098131.2017.1286368

Bensimon, M., Amir, D., & Wolf, Y. (2012). A pendulum between trauma and life: Group music therapy with post-traumatized soldiers. *Arts in Psychotherapy*, *39*(4 PG-223–233), 223–233. https://doi.org/10.1016/j.aip.2012.03.005

Bensimon, M., Amir, D., & Wolf, Y. (2008). Drumming through trauma: Music therapy with post-traumatic soldiers. *Arts in Psychotherapy*, *35*(1 PG-34–48), 34–48. https://doi.org/10.1016/j.aip.2007.09.002

Birks, M. (2007). Benefits of salsa classes in treatment of depression. *Nursing Times*, *103*(10 PG-32–33), 32–33. Retrieved from http://search.ebscohost.com/login.aspx?direct=true&db=rzh&AN=106283383&site=ehost-live&scope=site NS  -

Bittman, B., Dickson, L., & Coddington, K. (2009). Creative musical expression as a catalyst for quality-of-life improvement in inner-city adolescents placed in a court-referred residential treatment program. *Adv Mind Body Med*, *24*(1 PG-8–19), 8–19. Retrieved from NS  -

Blake, R. L. (1994). Vietnam veterans with post traumatic stress disorder: Findings from a music and imagery project. *Journal of the Association for Music and Imagery*, *3*(PG-5-18), 5–18. Retrieved from http://search.ebscohost.com/login.aspx?direct=true&db=rih&AN=A726775&site=ehost-live&scope=site NS  -

Blake, R. L., & Bishop, S. R. (1994). The Bonny Method of Guided Imagery and Music (GIM) in the treament of post-traumatic stress disorder (PTSD) with adults in the psychiatric setting. *Music Therapy Perspectives*, *12*(2 PG-125–129), 125–129. https://doi.org/10.1093/mtp/12.2.125

Blanaru, M., Bloch, B., Vadas, L., Arnon, Z., Ziv, N., Kremer, I., & Haimov, I. (2012). The effects of music relaxation and muscle relaxation techniques on sleep quality and emotional measures among individuals with posttraumatic stress disorder. *Ment Illn*, *4*(2 PG-e13), e13. https://doi.org/10.4081/mi.2012.e13

Bloch, B., Reshef, A., Vadas, L., Haliba, Y., Ziv, N., Kremer, I., & Haimov, I. (2010). The effects of music relaxation on sleep quality and emotional measures in people living with schizophrenia. *J Music Ther*, *47*(1 PG-27–52), 27–52. https://doi.org/10.1093/jmt/47.1.27

Bohnert, K. (1999). Meaningful musical experience and the treatment of an individual in psychosis: A case study. *Music Therapy Perspectives*, *17*(2 PG-69–73), 69–73. https://doi.org/10.1093/mtp/17.2.69

Bradford, D. L. (1991). Music as an adjunctive therapeutic mode in the treatment of depression. *Dissertation Abstracts International*, *51*(10‐B PG-5020‐5021), 5020‐5021. Retrieved from https://www.cochranelibrary.com/central/doi/10.1002/central/CN-00711559/full NS  -

Brakemeier, E. L., Engel, V., Schramm, E., Zobel, I., Schmidt, T., Hautzinger, M., … Normann, C. (2011). Feasibility and outcome of cognitive behavioral analysis system of psychotherapy (CBASP) for chronically depressed inpatients: A pilot study. *Psychotherapy and Psychosomatics*, *80*(3 PG-191–194), 191–194. https://doi.org/10.1159/000320779

Braun Janzen, T., Al Shirawi, M. I., Rotzinger, S., Kennedy, S. H., & Bartel, L. (2019). A Pilot Study Investigating the Effect of Music-Based Intervention on Depression and Anhedonia. *Front Psychol*, *10*(PG-1038), 1038. https://doi.org/10.3389/fpsyg.2019.01038

Buffum, M. D. (1993). Commentary on Dance movement: a therapeutic program for psychiatric clients [original article by Heber L appears in PERSPECT PSYCHIATR CARE 1993;29(2):22-9]. *AWHONN’s Women’s Health Nursing Scan*, *7*(6 PG-17–17), 17. Retrieved from http://search.ebscohost.com/login.aspx?direct=true&db=rzh&AN=107453018&site=ehost-live&scope=site NS  -

Campbell, B. (2019). Past, Present, Future: A Program Development Project Exploring Post Traumatic Slave Syndrome (PTSS) Using Experiential Education and Dance/Movement Therapy Informed Approaches. *American Journal of Dance Therapy*, *41*(2 PG-214–233), 214–233. https://doi.org/10.1007/s10465-019-09320-8

Carr, C., d’Ardenne, P., Sloboda, A., Scott, C., Wang, D., & Priebe, S. (2012). Group music therapy for patients with persistent post-traumatic stress disorder--an exploratory randomized controlled trial with mixed methods evaluation. *Psychol Psychother*, *85*(2 PG-179–202), 179–202. https://doi.org/10.1111/j.2044-8341.2011.02026.x

Carroll, M. L. (2019). *The initiation of receptive sound engagement with a female adult survivor of childhood sexual abuse: An archival clinical case study*. ProQuest Information & Learning. Retrieved from http://search.ebscohost.com/login.aspx?direct=true&db=psyh&AN=2018-58621-108&site=ehost-live&scope=site NS  -

Castelino, A. (2009). *The effect of single sessions of music therapy on the level of anxiety in older persons with psychiatric disorders: A pilot study*. Retrieved from http://search.ebscohost.com/login.aspx?direct=true&db=rih&AN=A841836&site=ehost-live&scope=site http://researcharchive.vuw.ac.nz/bitstream/handle/10063/1052/thesis.pdf?sequence=1 NS  -

Ceccato, E., Caneva, P., & Lamonaca, D. (2006). Music therapy and cognitive rehabilitation in schizophrenic patients: a controlled study. *Nordic Journal of Music Therapy*, *15*(2 PG-111–120), 111–120. Retrieved from http://search.ebscohost.com/login.aspx?direct=true&db=rzh&AN=106297578&site=ehost-live&scope=site NS  -

Cercone, K.A. (2007). The effects of music therapy on symptoms of schizophrenia and other serious mental illnesses: A meta-analysis. *Dissertation Abstracts International: Section B: The Sciences and Engineering*, *68*(9-B). Retrieved from https://www.epistemonikos.org/en/documents/bb5ea019921d7adfdfb94accfbd72545fd2d0ade

Chan, M. F., Wong, Z. Y., Onishi, H., & Thayala, N. V. (2012). Effects of music on depression in older people: a randomised controlled trial. *J Clin Nurs*, *21*(5-6 PG-776–83), 776–783. https://doi.org/10.1111/j.1365-2702.2011.03954.x

Chan, M. F., Wong, Z. Y., & Thayala, N. V. (2011). The effectiveness of music listening in reducing depressive symptoms in adults: a systematic review. *Complement Ther Med*, *19*(6 PG-332–48), 332–348. https://doi.org/10.1016/j.ctim.2011.08.003

Chan, M. F., Wong, Z. Y., & Thayala, N. V. (2010). A systematic review on the effectiveness of music listening in reducing depressive symptoms in adults. *JBI Libr Syst Rev*, *8*(31 PG-1242–1287), 1242–1287. https://doi.org/10.11124/01938924-201008310-00001

Chang, B. H., Chen, B. W., Beckstead, J. W., & Yang, C. Y. (2018). Effects of a music-creation programme on the anxiety, self-esteem, and quality of life of people with severe mental illness: A quasi-experimental design. *Int J Ment Health Nurs*, *27*(3 PG-1066–1076), 1066–1076. https://doi.org/10.1111/inm.12414

Chen, C. J., Sung, H. C., Lee, M. S., & Chang, C. Y. (2015). The effects of Chinese five-element music therapy on nursing students with depressed mood. *Int J Nurs Pract*, *21*(2 PG-192–9), 192–199. https://doi.org/10.1111/ijn.12236

Chen, M. D., Kuo, Y. H., Chang, Y. C., Hsu, S. T., Kuo, C. C., & Chang, J. J. (2016). Influences of Aerobic Dance on Cognitive Performance in Adults with Schizophrenia. *Occup Ther Int*, *23*(4 PG-346–356), 346–356. https://doi.org/10.1002/oti.1436

Chen, Y. J., Li, X. X., Pan, B., Wang, B., Jing, G. Z., Liu, Q. Q., … Ge, L. (2019). Non-pharmacological interventions for older adults with depressive symptoms: a network meta-analysis of 35 randomized controlled trials. *Aging Ment Health*, (PG-1-14), 1–14. https://doi.org/10.1080/13607863.2019.1704219

Chiang, M., Reid-Varley, W. B., & Fan, X. (2019, May 1). Creative art therapy for mental illness. *Psychiatry Research*. Elsevier Ireland Ltd. https://doi.org/10.1016/j.psychres.2019.03.025

Choi, C. M. H. (2008). *The effects of the modified CARING at Columbia Music Therapy program on the psychological conditions of refugee adolescents from North Korea*. ProQuest Information & Learning. Retrieved from http://search.ebscohost.com/login.aspx?direct=true&db=psyh&AN=2008-99091-053&site=ehost-live&scope=site NS  -

Chou, M. H., & Lin, M. F. (2006). Exploring the listening experiences during guided imagery and music therapy of outpatients with depression. *J Nurs Res*, *14*(2 PG-93–102), 93–102. https://doi.org/10.1097/01.jnr.0000387567.41941.14

Christrup, H. J. (1962). The effect of dance therapy on the concept of body image. *Psychiatric Quarterly Supplement*, *36*(2 PG-296–303), 296–303. Retrieved from http://search.ebscohost.com/login.aspx?direct=true&db=psyh&AN=1965-02139-001&site=ehost-live&scope=site NS  -

Chu, H., Yang, C. Y., Lin, Y., Ou, K. L., Lee, T. Y., O’Brien, A. P., & Chou, K. R. (2014). The impact of group music therapy on depression and cognition in elderly persons with dementia: a randomized controlled study. *Biol Res Nurs*, *16*(2 PG-209–17), 209–217. https://doi.org/10.1177/1099800413485410

Chung, J., & Woods-Giscombe, C. (2016). Influence of Dosage and Type of Music Therapy in Symptom Management and Rehabilitation for Individuals with Schizophrenia. *Issues Ment Health Nurs*, *37*(9 PG-631–641), 631–641. https://doi.org/10.1080/01612840.2016.1181125

Cook, B. J. (2016). *Utilizing music within a school environment to reduce anxiety in an adolescent female*. ProQuest Information & Learning. Retrieved from http://search.ebscohost.com/login.aspx?direct=true&db=psyh&AN=2016-26527-283&site=ehost-live&scope=site NS  -

Cook, M., & Freethy, M. (1973). The use of music as a positive reinforcer to eliminate complaining behavior. *Journal of Music Therapy*, *10*(4 PG-213–216), 213–216. https://doi.org/10.1093/jmt/10.4.213

Covington, H., & Crosby, C. (1997). Music therapy as a nursing intervention. *J Psychosoc Nurs Ment Health Serv*, *35*(3 PG-34–7), 34–37. Retrieved from NS  -

Curreri, E. (2015). An Unguided Music Therapy Listening Experience of Luigi Nono’s Fragmente-Stille, an Diotima: A Case Report. *Music Therapy Perspectives*, *33*(1 PG-63–70), 63–70. https://doi.org/10.1093/mtp/miu047

Curreri, E. (2013). Aesthetic Perturbation: Using a Chance/Aleatoric Music Therapy Intervention to Reduce Rigidity in Adult Patients with Psychiatric Disorders. *Music Therapy Perspectives*, *31*(2 PG-105–111), 105–111. https://doi.org/10.1093/mtp/31.2.105

Curreri, E. (2017). Connecting to Self and Other through Electroacoustic Composition and Performance: A Case Study. *Music Therapy Perspectives*, *35*(2 PG-209–218), 209–218. https://doi.org/10.1093/mtp/miw007

Davies, A. (1995). The acknowledgment of loss working through depression. *British Journal of Music Therapy*, *9*(1 PG-11–16), 11–16. Retrieved from http://search.ebscohost.com/login.aspx?direct=true&db=rih&AN=A560124&site=ehost-live&scope=site NS  -

de Niet, G., Tiemens, B., & Hutschemaekers, G. (2010). Can mental healthcare nurses improve sleep quality for inpatients? *Br J Nurs*, *19*(17 PG-1100–5), 1100–1105. https://doi.org/10.12968/bjon.2010.19.17.78558

Deatrich, K. G., Prout, M. F., Boyer, B. A., & Yoder, S. E. (2016). Effectiveness of group music therapy in a psychiatric hospital: A randomized pilot study of treatment outcome. *International Journal of Group Psychotherapy*, *66*(4 PG-592–617), 592–617. https://doi.org/10.1080/00207284.2016.1190239

Degli Stefani, M., & Biasutti, M. (2016). Effects of Music Therapy on Drug Therapy of Adult Psychiatric Outpatients: A Pilot Randomized Controlled Study. *Front Psychol*, *7*(PG-1518), 1518. https://doi.org/10.3389/fpsyg.2016.01518

Deshmukh, A. D., Sarvaiya, A. A., Seethalakshmi, R., & Nayak, A. S. (2009). Effect of Indian classical music on quality of sleep in depressed patients: A randomized controlled trial. *Nordic Journal of Music Therapy*, *18*(1 PG-70–78), 70–78. https://doi.org/10.1080/08098130802697269

Dickinson, S. C. (2012). A case of work, rest and play: Music therapy in early onset psychosis. In *Collected Work: Forensic music therapy: A treatment for men and women in secure hospital settings. Published by: Jessica Kingsley, London, Great Britain, 2012; Published by: Jessica Kingsley, Philadelphia, PA, United States, 2013. Pages: 89-103. (AN: 2012*. London Philadelphia: Jessica Kingsley. Retrieved from http://search.ebscohost.com/login.aspx?direct=true&db=rih&AN=A848339&site=ehost-live&scope=site NS  -

Dimiceli-Mitran, L. (2017). Accessing strength from within: Music imagery and mandalas. In *Handbook of strengths-based clinical practices: Finding common factors.* (pp. 164–178). New York, NY: Routledge/Taylor & Francis Group. Retrieved from http://search.ebscohost.com/login.aspx?direct=true&db=psyh&AN=2016-60501-011&site=ehost-live&scope=site NS  -

Dingle, G. A., Williams, E., Jetten, J., & Welch, J. (2017). Choir singing and creative writing enhance emotion regulation in adults with chronic mental health conditions. *Br J Clin Psychol*, *56*(4 PG-443–457), 443–457. https://doi.org/10.1111/bjc.12149

Doerksen, C. (1997). *The use of relaxation training and biofeedback to increase human approach responses in individuals with schizophrenia*. ProQuest Information & Learning. Retrieved from http://search.ebscohost.com/login.aspx?direct=true&db=psyh&AN=1997-95008-015&site=ehost-live&scope=site NS  -

Dunphy, K., Baker, F. A., Dumaresq, E., Carroll-Haskins, K., Eickholt, J., Ercole, M., … Wosch, T. (2019). Creative arts interventions to address depression in older adults: A systematic review of outcomes, processes, and mechanisms. *Frontiers in Psychology*, *9*(PG-). https://doi.org/10.3389/fpsyg.2018.02655

Dvorkin, J. (1982). Piano improvisation: A therapeutic tool in acceptance and resolution of emotions in a schizo-affective personality. *Music Therapy*, *2*(1 PG-53–62), 53–62. https://doi.org/10.1093/mt/2.1.53

Edwards, J. (2006). Music therapy in the treatment and management of mental disorders. *Irish Journal of Psychological Medicine*, *23*(1), 33–35. https://doi.org/10.1017/S0790966700009459

Egenti, N. T., Ede, M. O., Nwokenna, E. N., Oforka, T., Nwokeoma, B. N., Mezieobi, D. I., … Victor-Aigbodion, V. (2019). Randomized controlled evaluation of the effect of music therapy with cognitive-behavioral therapy on social anxiety symptoms. *Medicine (Baltimore)*, *98*(32 PG-e16495), e16495. https://doi.org/10.1097/md.0000000000016495

Elizabeth Carr, C., O’Kelly, J., Sandford, S., Priebe, S., & Carr, C. E. (2017). Feasibility and acceptability of group music therapy vs wait-list control for treatment of patients with long-term depression (the SYNCHRONY trial): study protocol for a randomised controlled trial. *Trials*, *18*(PG-1-15), 1–15. https://doi.org/10.1186/s13063-017-1893-8

Erkkila, J., Punkanen, M., Fachner, J., Ala-Ruona, E., Pontio, I., Tervaniemi, M., … Gold, C. (2011). Individual music therapy for depression: randomised controlled trial. *Br J Psychiatry*, *199*(2 PG-132–9), 132–139. https://doi.org/10.1192/bjp.bp.110.085431

Ertekin Pinar Rn, P. S., & Tel, R. N. P. H. (2018). The effect of music on auditory hallucination and quality of life in schizophrenic patients: A randomised controlled trial. *Issues in Mental Health Nursing*, (PG-). https://doi.org/10.1080/01612840.2018.1463324

Ertekin Pinar S Rn, P., & Tel H Rn, P. (2019). The Effect of Music on Auditory Hallucination and Quality of Life in Schizophrenic Patients: A Randomised Controlled Trial. *Issues Ment Health Nurs*, *40*(1 PG-50–57), 50–57. https://doi.org/10.1080/01612840.2018.1463324

Esfandiari, N., & Mansouri, S. (2014). The effect of listening to light and heavy music on reducing the symptoms of depression among female students. *Arts in Psychotherapy*, *41*(2 PG-211–213), 211–213. https://doi.org/10.1016/j.aip.2014.02.001

Esparza, J. (2016). *Using person-centered expressive arts group therapy with combat-related PTSD Veterans*. ProQuest Information & Learning. Retrieved from http://search.ebscohost.com/login.aspx?direct=true&db=psyh&AN=2016-31155-090&site=ehost-live&scope=site NS  -

Eyre, L. (2008). *Musically-prepared and non musically-prepared narratives by persons living with schizophrenia*. Temple University. Retrieved from http://search.ebscohost.com/login.aspx?direct=true&db=rzh&AN=109850838&site=ehost-live&scope=site NS  -

Eyre, L. (2011). From ego disintegration to recovery of self: The contribution of Lacan’s theories in understanding the role of music therapy in the treatment of a woman with psychosis. In *Collected Work: Developments in music therapy practice: Case study perspectives. Published by: Barcelona, Gilsum, NH, U.S.A, 2011. Pages: 385-399. (AN: 2011-14783).* Gilsum: Barcelona. Retrieved from http://search.ebscohost.com/login.aspx?direct=true&db=rih&AN=A805369&site=ehost-live&scope=site NS  -

Eyre, L. (2003). Into the Labyrinth: Music Therapy in the Treatment of an Individual with Chronic Schizophrenia. *Canadian Journal of Music Therapy*, *10*(1 PG-8–32), 8–32. Retrieved from http://search.ebscohost.com/login.aspx?direct=true&db=psyh&AN=2005-08328-002&site=ehost-live&scope=site NS  -

Fancourt, D., & Perkins, R. (2018). Effect of singing interventions on symptoms of postnatal depression: three-arm randomised controlled trial. *Br J Psychiatry*, *212*(2 PG-119–121), 119–121. https://doi.org/10.1192/bjp.2017.29

Felsenstein, R. (2013). From uprooting to replanting: on post-trauma group music therapy for pre-school children. *Nordic Journal of Music Therapy*, *22*(1 PG-69–85), 69–85. https://doi.org/10.1080/08098131.2012.667824

Feng, K., Shen, C. Y., Ma, X. Y., Chen, G. F., Zhang, M. L., Xu, B., … Ju, Y. (2019). Effects of music therapy on major depressive disorder: A study of prefrontal hemodynamic functions using fNIRS. *Psychiatry Res*, *275*(PG-86-93), 86–93. https://doi.org/10.1016/j.psychres.2019.03.015

Finston, P. (2009). Extraordinary meridian treatment for severe mental disorders plus augmentation with psychotherapy, Indian Raga music, and essential oils. *Medical Acupuncture*, *21*(1 PG-27–34), 27–34. https://doi.org/10.1089/acu.2009.0644

Fitch, L., & LaRoche, R. (1985). Earth House: An alternative for persons suffering from the schizophrenias. *Journal of Orthomolecular Psychiatry*, *14*(2 PG-136–142), 136–142. Retrieved from http://search.ebscohost.com/login.aspx?direct=true&db=psyh&AN=1986-17718-001&site=ehost-live&scope=site NS  -

Frank, G. (2005). David’s music: an observation of musical interactions with a schizophrenic adolescent. *Nordic Journal of Music Therapy*, *14*(2 PG-179–185), 179–185. Retrieved from http://search.ebscohost.com/login.aspx?direct=true&db=rzh&AN=106413804&site=ehost-live&scope=site NS  -

Freedman, A. M. (1959). Day hospitals for severely disturbed schizophrenic children. *American Journal of Psychiatry*, *115*(10 PG-893–898), 893–898. Retrieved from http://www.embase.com/search/results?subaction=viewrecord&from=export&id=L280632478 NS  -

Friedman, S. H., Kaplan, R. S., Rosenthal, M. B., & Console, P. (2010). Music therapy in perinatal psychiatry: Use of lullabies for pregnant and postpartum women with mental illness. *Music and Medicine*, *2*(4 PG-219–225), 219–225. https://doi.org/10.1177/1943862110379584

Gallagher, A. G., Dinan, T. G., & Baker, L. J. (1994). The effects of varying auditory input on schizophrenic hallucinations: a replication. *Br J Med Psychol*, *67 ( Pt 1)*(PG-67-75), 67–75. https://doi.org/10.1111/j.2044-8341.1994.tb01771.x

Gao, L., Zhang, L., Qi, H., & Petridis, L. (2016). Middle-aged Female Depression in Perimenopausal Period and Square Dance Intervention. *Psychiatr Danub*, *28*(4 PG-372–378), 372–378. Retrieved from NS  -

Gao, T. (2013). An Introduction to MER, a New Music Psychotherapy Approach for PTSD: Part 2—The Outcomes and Case Examples. *Music & Medicine*, *5*(2 PG-105–109), 105–109. https://doi.org/10.1177/1943862113487383

Garrido, S., Eerola, T., & McFerran, K. (2017). Group Rumination: Social Interactions Around Music in People with Depression. *Front Psychol*, *8*(PG-490), 490. https://doi.org/10.3389/fpsyg.2017.00490

Gavrielidou, M., & Odell-Miller, H. (2017). An investigation of pivotal moments in music therapy in adult mental health. *Arts in Psychotherapy*, *52*(PG-50-62), 50–62. https://doi.org/10.1016/j.aip.2016.09.006

Gay, K., Torous, J., Joseph, A., Pandya, A., & Duckworth, K. (2016). Digital Technology Use Among Individuals with Schizophrenia: Results of an Online Survey. *JMIR Ment Health*, *3*(2 PG-e15), e15. https://doi.org/10.2196/mental.5379

Gebhardt, S., Dammann, I., Loescher, K., Wehmeier, P. M., Vedder, H., & von Georgi, R. (2018). The effects of music therapy on the interaction of the self and emotions-An interim analysis. *Complement Ther Med*, *41*(PG-61-66), 61–66. https://doi.org/10.1016/j.ctim.2018.08.014

Gebhardt, S., & von Georgi, R. (2015). The Change of Music Preferences Following the Onset of a Mental Disorder. *Ment Illn*, *7*(1 PG-5784), 5784. https://doi.org/10.4081/mi.2015.5784

Geipel, J., Koenig, J., Hillecke, T. K., Resch, F., & Kaess, M. (2018). Music-based interventions to reduce internalizing symptoms in children and adolescents: A meta-analysis. *J Affect Disord*, *225*(PG-647-656), 647–656. https://doi.org/10.1016/j.jad.2017.08.035

Gerber, M. M., Hogan, L. R., Maxwell, K., Callahan, J. L., Ruggero, C. J., & Sundberg, T. (2014). Children after war: A novel approach to promoting resilience through music. *Traumatology: An International Journal*, *20*(2 PG-112–118), 112–118. https://doi.org/10.1037/h0099396

Geretsegger, M., Mössler, K., Bieleninik, Ł., Chen, X., Heldal, T. O., & Gold, C. (2017). Music therapy for people with schizophrenia and schizophrenia-like disorders. *Cochrane Database of Systematic Reviews*, (5 PG-102–102), 102. Retrieved from http://search.ebscohost.com/login.aspx?direct=true&db=rih&AN=A1262188&site=ehost-live&scope=site NS  -

Gillis, A., Lascelles, C. F., & Crone, N. (1958). A comparison of rhythmic and non-rhythmic music in chronic schizophrenia. *Am J Psychiatry*, *114*(12 PG-1111–2), 1111–1112. https://doi.org/10.1176/ajp.114.12.1111-a

Glicksohn, J., & Cohen, Y. (2000). Can music alleviate cognitive dysfunction in schizophrenia? *Psychopathology*, *33*(1 PG-43–7), 43–47. https://doi.org/10.1159/000029118

Goertzel, V., May, P. R., Salkin, J., & Schoop, T. (1965). Body-ego technique: An approach to the schizophrenic patient. *Journal of Nervous and Mental Disease*, *141*(1 PG-53–60), 53–60. https://doi.org/10.1097/00005053-196507000-00005

Gok Ugur, H., Yaman Aktas, Y., Orak, O. S., Saglambilen, O., & Aydin Avci, I. (2017). The effect of music therapy on depression and physiological parameters in elderly people living in a Turkish nursing home: a randomized-controlled trial. *Aging Ment Health*, *21*(12 PG-1280–1286), 1280–1286. https://doi.org/10.1080/13607863.2016.1222348

Gold, C., Eickholt, J., Assmus, J., Stige, B., Wake, J. D., Baker, F. A., … Geretsegger, M. (2019). Music Interventions for Dementia and Depression in ELderly care (MIDDEL): protocol and statistical analysis plan for a multinational cluster-randomised trial. *BMJ Open*, *9*(3 PG-e023436), e023436. https://doi.org/10.1136/bmjopen-2018-023436

Gold, C., Heldal, T. O., Dahle, T., & Wigram, T. (2005). Music therapy for schizophrenia or schizophrenia-like illnesses. *Cochrane Database Syst Rev*, (2 PG-Cd004025), Cd004025. https://doi.org/10.1002/14651858.CD004025.pub2

Gold, C., Mossler, K., Grocke, D., Heldal, T. O., Tjemsland, L., Aarre, T., … Rolvsjord, R. (2013). Individual music therapy for mental health care clients with low therapy motivation: multicentre randomised controlled trial. *Psychother Psychosom*, *82*(5 PG-319–31), 319–331. https://doi.org/10.1159/000348452

Gold, C., Saarikallio, S., Crooke, A. H. D., & McFerran, K. S. (2017). Group Music Therapy as a Preventive Intervention for Young People at Risk: Cluster-Randomized Trial. *J Music Ther*, *54*(2 PG-133–160), 133–160. https://doi.org/10.1093/jmt/thx002

Gold, C., Dahle, T., Heldal, T. O., & Wigram, T. (2006). Music therapy for people with schizophrenia or other psychoses: A systematic review and meta-analysis. *British Journal of Music Therapy*, *20*(2 PG-100–108), 100–108. Retrieved from http://search.ebscohost.com/login.aspx?direct=true&db=rih&AN=A584787&site=ehost-live&scope=site NS  -

Gold, C., Solli, H. P., Krüger, V., & Lie, S. A. (2009, April). Dose-response relationship in music therapy for people with serious mental disorders: Systematic review and meta-analysis. *Clinical Psychology Review*. https://doi.org/10.1016/j.cpr.2009.01.001

Goldbeck, L., & Ellerkamp, T. (2012). A randomized controlled trial of multimodal music therapy for children with anxiety disorders. *J Music Ther*, *49*(4 PG-395–413), 395–413. https://doi.org/10.1093/jmt/49.4.395

Goldstein-Roca, S., & Crisafulli, T. (1994). Integrative creative arts therapy: A brief treatment model. *The Arts in Psychotherapy*, *21*(3 PG-219–222), 219–222. https://doi.org/10.1016/0197-4556(94)90051-5

Gooding, L. F., & Langston, D. G. (2019). Music Therapy With Military Populations: A Scoping Review. *Journal of Music Therapy*, *56*(4), 315–347. https://doi.org/10.1093/jmt/thz010

Gooding, L. F. (2011). *The effect of a music therapy-based social skills training program on social competence in children and adolescents with social skills deficits*. ProQuest Information & Learning. Retrieved from http://search.ebscohost.com/login.aspx?direct=true&db=psyh&AN=2011-99030-282&site=ehost-live&scope=site NS  -

Gorden-Cohen, N. (1987). Vietnam and reality: The story of Mr D. *American Journal of Dance Therapy*, *10*(PG-95-109), 95–109. Retrieved from http://search.ebscohost.com/login.aspx?direct=true&db=psyh&AN=1997-72082-001&site=ehost-live&scope=site NS  -

Gosselin, Ém., Lavoie, S., Bourgault, P., & GÉLinas, Cél. (2019). Intervention for patients intubated and conscious to decrease peritraumatic distress (IPIC-PTD)--Preliminary results. *Canadian Journal of Critical Care Nursing*, *30*(1 PG-32–42), 32–42. Retrieved from http://search.ebscohost.com/login.aspx?direct=true&db=rzh&AN=135289198&site=ehost-live&scope=site NS  -

Grasser, L. R., Al-Saghir, H., Wanna, C., Spinei, J., & Javanbakht, A. (2019). Moving Through the Trauma: Dance/Movement Therapy as a Somatic-Based Intervention for Addressing Trauma and Stress Among Syrian Refugee Children. *J Am Acad Child Adolesc Psychiatry*, *58*(11 PG-1124–1126), 1124–1126. https://doi.org/10.1016/j.jaac.2019.07.007

Grebosz-Haring, K., & Thun-Hohenstein, L. (2018). Effects of group singing versus group music listening on hospitalized children and adolescents with mental disorders: A pilot study. *Heliyon*, *4*(12 PG-e01014), e01014. https://doi.org/10.1016/j.heliyon.2018.e01014

Green, A. (2011). Art and Music Therapy for Trauma Survivors. *Canadian Art Therapy Association Journal*, *24*(2 PG-14–19), 14–19. Retrieved from http://search.ebscohost.com/login.aspx?direct=true&db=rzh&AN=108203644&site=ehost-live&scope=site NS  -

Grocke, D., Bloch, S., & Castle, D. (2009). The effect of group music therapy on quality of life for participants living with a severe and enduring mental illness. *J Music Ther*, *46*(2 PG-90–104), 90–104. https://doi.org/10.1093/jmt/46.2.90

Grocke, D., Bloch, S., Castle, D., Thompson, G., Newton, R., Stewart, S., & Gold, C. (2014). Group music therapy for severe mental illness: a randomized embedded-experimental mixed methods study. *Acta Psychiatr Scand*, *130*(2 PG-144–53), 144–153. https://doi.org/10.1111/acps.12224

Grocke, D., Bloch, S., & Castle, D. (2008). Is there a role for music therapy in the care of the severely mentally ill? *Australasian Psychiatry*, *16*(6), 442–445. https://doi.org/10.1080/10398560802366171

Grodner, S., Braff, D. L., Janowsky, D. S., & Clopton, P. L. (1982). Efficacy of art/movement therapy in elevating mood. *The Arts in Psychotherapy*, *9*(3 PG-217–225), 217–225. https://doi.org/10.1016/0197-4556(82)90038-7

Guetin, S., Soua, B., Voiriot, G., Picot, M. C., & Herisson, C. (2009). The effect of music therapy on mood and anxiety-depression: an observational study in institutionalised patients with traumatic brain injury. *Ann Phys Rehabil Med*, *52*(1 PG-30–40), 30–40. https://doi.org/10.1016/j.annrmp.2008.08.009

Gunning, S. V, & Holmes, T. H. (1973). Dance therapy with psychotic children. Definition and quantitative evaluation. *Archives of General Psychiatry*, *28*(5 PG-707–713), 707–713. Retrieved from http://www.embase.com/search/results?subaction=viewrecord&from=export&id=L93349567 NS  -

Gutiérrez, E. O. F., & Camarena, V. A. T. (2015). Music therapy in generalized anxiety disorder. *Arts in Psychotherapy*, *44*(PG-19-24), 19–24. https://doi.org/10.1016/j.aip.2015.02.003

Haboush, A., Floyd, M., Caron, J., LaSota, M., & Alvarez, K. (2006). Ballroom dance lessons for geriatric depression: An exploratory study. *Arts in Psychotherapy*, *33*(2 PG-89–97), 89–97. https://doi.org/10.1016/j.aip.2005.10.001

Hackney, M. E., & Earhart, G. M. (2010). Social partnered dance for people with serious and persistent mental illness: a pilot study. *J Nerv Ment Dis*, *198*(1 PG-76–8), 76–78. https://doi.org/10.1097/NMD.0b013e3181c81f7c

Haimov, I., Blanaro, M., Arnon, Z., Ziv, N., Bloch, B., Reshef, A., … Kremer, I. (2010). The effects of music and muscle relaxation therapies on sleep quality in individuals with post-traumatic stress disorder. *Journal of Sleep Research*, *19*(PG-364), 364. https://doi.org/10.1111/j.1365-2869.2010.00868.x

Haller, H., Anheyer, D., Cramer, H., & Dobos, G. (2019). Complementary therapies for clinical depression: An overview of systematic reviews. *BMJ Open*, *9*(8 PG-). https://doi.org/10.1136/bmjopen-2018-028527

Hamer, B. A. (1991). Music therapy: harmony for change. *J Psychosoc Nurs Ment Health Serv*, *29*(12 PG-5–7), 5–7. Retrieved from NS  -

Hannibal, N., Pedersen, I. N., Hestbaek, T., Sorensen, T. E., & Munk-Jorgensen, P. (2012). Schizophrenia and personality disorder patients’ adherence to music therapy. *Nord J Psychiatry*, *66*(6 PG-376–9), 376–379. https://doi.org/10.3109/08039488.2012.655775

Hannibal, N., Xu, K., Gold, C., & Chen, X. J. (2014). Group Music Therapy for Prisoners: Protocol for a Randomized Controlled Trial. *Nordic Journal of Music Therapy*, *23*(3 PG-224–241), 224–241. Retrieved from https://search.proquest.com/docview/1586011199?accountid=11752 http://findit.library.jhu.edu/resolve?url_ver=Z39.88-2004&rft_val_fmt=info:ofi/fmt:kev:mtx:journal&genre=article&sid=ProQ:ProQ%3Aiimpft&atitle=Group+Music+Therapy+for+Prisoners%3A+Protocol+for

Hanser, S. B. (1990). A music therapy strategy for depressed older adults in the community. *Journal of Applied Gerontology*, *9*(3 PG-283–298), 283–298. Retrieved from http://www.embase.com/search/results?subaction=viewrecord&from=export&id=L20354837 NS  -

Hanser, S. B., & Thompson, L. W. (1994). Effects of a music therapy strategy on depressed older adults. *J Gerontol*, *49*(6 PG-P265-9), P265-9. https://doi.org/10.1093/geronj/49.6.p265

Hauck, L. P., & Martin, P. L. (1970). Music as a reinforcer in patient-controlled duration of time-out. *Journal of Music Therapy*, *7*(2 PG-43–53), 43–53. https://doi.org/10.1093/jmt/7.2.43

Hayashi, N., Tanabe, Y., Nakagawa, S., Noguchi, M., Iwata, C., Koubuchi, Y., … Koike, I. (2002). Effects of group musical therapy on inpatients with chronic psychoses: a controlled study. *Psychiatry Clin Neurosci*, *56*(2 PG-187–93), 187–193. https://doi.org/10.1046/j.1440-1819.2002.00953.x

He, H., Yang, M., Duan, M., Chen, X., Lai, Y., Xia, Y., … Yao, D. (2018). Music intervention leads to increased insular connectivity and improved clinical symptoms in schizophrenia. *Frontiers in Neuroscience*, *11*(JAN PG-). https://doi.org/10.3389/fnins.2017.00744

Heber, L. (1993). Ethnic/movement dance: therapeutic intervention for psychiatric clients. *Concern*, *22*(5 PG-20–1), 20–21. Retrieved from NS  -

Heiderscheit, A., & Madson, A. M. Y. (2015). Use of the Iso Principle as a Central Method in Mood Management: A Music Psychotherapy Clinical Case Study. *Music Therapy Perspectives*, *33*(1 PG-45–52), 45–52. https://doi.org/10.1093/mtp/miu042

Helgason, C., & Sarris, J. (2013, October). Mind-body medicine for schizophrenia and psychotic disorders: A review of the evidence. *Clinical Schizophrenia and Related Psychoses*. https://doi.org/10.3371/CSRP.HESA.020813

Hense, C., McFerran, K. S., & McGorry, P. (2014). Constructing a grounded theory of young people’s recovery of musical identity in mental illness. *Arts in Psychotherapy*, *41*(5 PG-594–603), 594–603. https://doi.org/10.1016/j.aip.2014.10.010

Hernandez-Ruiz, E. (2005). Effect of music therapy on the anxiety levels and sleep patterns of abused women in shelters. *J Music Ther*, *42*(2 PG-140–58), 140–158. https://doi.org/10.1093/jmt/42.2.140

Hitchen, H., Magee, W. L., & Soeterik, S. (2010). Music therapy in the treatment of patients with neuro-behavioural disorders stemming from acquired brain injury. *Nordic Journal of Music Therapy*, *19*(1 PG-63–78), 63–78. https://doi.org/10.1080/08098130903086404

Hovey, S. A. (2013). The Effects of Musical Activity on the Self-Esteem and Self-Efficacy of Patients With Schizophrenia: A Cultural Study in West Bengal, India. *Music & Medicine*, *5*(1 PG-NP1-4), NP1-4. https://doi.org/10.1177/1943862112467465

Hsu, W. C., & Lai, H. L. (2004). Effects of music on major depression in psychiatric inpatients. *Arch Psychiatr Nurs*, *18*(5 PG-193–9), 193–199. https://doi.org/10.1016/j.apnu.2004.07.007

Hustig, H. H., Tran, D. B., Hafner, R. J., & Miller, R. J. (1990). The effect of headphone music on persistent auditory hallucinations. *Behavioural Psychotherapy*, *18*(4 PG-273–281), 273–281. Retrieved from http://www.embase.com/search/results?subaction=viewrecord&from=export&id=L21046435 NS  -

Hylton, E., Malley, A., & Ironson, G. (2019). Improvements in adolescent mental health and positive affect using creative arts therapy after a school shooting: A pilot study. *Arts in Psychotherapy*, *65*(PG-). https://doi.org/10.1016/j.aip.2019.101586

Ihalainen, O., & Rosberg, G. (1976). Relaxing and encouraging suggestions given to hospitalized chronic schizophrenics. *Int J Clin Exp Hypn*, *24*(3 PG-228–37), 228–237. https://doi.org/10.1080/00207147608416204

Jenkins, L. M., Skerrett, K. A., DelDonno, S. R., Patron, V. G., Meyers, K. K., Peltier, S., … Starkman, M. N. (2018). Individuals with more severe depression fail to sustain nucleus accumbens activity to preferred music over time. *Psychiatry Res Neuroimaging*, *275*(PG-21-27), 21–27. https://doi.org/10.1016/j.pscychresns.2018.03.002

Jensen, B. (2005). The principle of polarisation used in working with schizophrenic patients. *Collected Work: Many Faces of Music Therapy. (AN: 2005-08385).*, *6*(4 PG-612–626), 612–626. Retrieved from http://search.ebscohost.com/login.aspx?direct=true&db=rih&AN=A511266&site=ehost-live&scope=site http://www.wfmt.info/Musictherapyworld/modules/archive/stuff/reports/Proceedings_Screen.pdf NS  -

Jeon, G. S., Gang, M., & Oh, K. (2017). The Effectiveness of the Nanta-Program on Psychiatric Symptoms, Interpersonal Relationships, and Quality of Life in Forensic Inpatients with Schizophrenia. *Arch Psychiatr Nurs*, *31*(1 PG-93–98), 93–98. https://doi.org/10.1016/j.apnu.2016.09.005

Jespersen, K. V, & Vuust, P. (2012). The Effect of Relaxation Music Listening on Sleep Quality in Traumatized Refugees: A Pilot Study. *J Music Ther*, *49*(2 PG-205–29), 205–229. https://doi.org/10.1093/jmt/49.2.205

Jorm, A. F., Christensen, H., Griffiths, K. M., Parslow, R. A., Rodgers, B., & Blewitt, K. A. (2004). Effectiveness of complementary and self-help treatments for anxiety disorders. *Med J Aust*, *181*(S7 PG-S29-46), S29-46. Retrieved from https://onlinelibrary.wiley.com/doi/full/10.5694/j.1326-5377.2004.tb06352.x?sid=nlm%3Apubmed NS  -

Kaelen, M., Giribaldi, B., Raine, J., Evans, L., Timmerman, C., Rodriguez, N., … Carhart-Harris, R. (2018). The hidden therapist: evidence for a central role of music in psychedelic therapy. *Psychopharmacology (Berl)*, *235*(2 PG-505–519), 505–519. https://doi.org/10.1007/s00213-017-4820-5

Kaltsatou, A., Kouidi, E., Fountoulakis, K., Sipka, C., Theochari, V., Kandylis, D., & Deligiannis, A. (2015). Effects of exercise training with traditional dancing on functional capacity and quality of life in patients with schizophrenia: a randomized controlled study. *Clin Rehabil*, *29*(9 PG-882–91), 882–891. https://doi.org/10.1177/0269215514564085

Karkou, V., Aithal, S., Zubala, A., & Meekums, B. (2019). Effectiveness of Dance Movement Therapy in the Treatment of Adults With Depression: A Systematic Review With Meta-Analyses. *Front Psychol*, *10*(PG-936), 936. https://doi.org/10.3389/fpsyg.2019.00936

Kavak, F., Unal, S., & Yilmaz, E. (2016). Effects of Relaxation Exercises and Music Therapy on the Psychological Symptoms and Depression Levels of Patients with Schizophrenia. *Arch Psychiatr Nurs*, *30*(5 PG-508–12), 508–512. https://doi.org/10.1016/j.apnu.2016.05.003

Kellett, S., Hall, J., & Compton Dickinson, S. (2019). Group cognitive analytic music therapy: a quasi-experimental feasibility study conducted in a high secure hospital. *Nordic Journal of Music Therapy*, *28*(3 PG-224–255), 224–255. https://doi.org/http://dx.doi.org/10.1080/08098131.2018.1529697

Kelly, B. S. (2001). *Psychophysiological responses to classical, new age, and Native American music*. ProQuest Information & Learning. Retrieved from http://search.ebscohost.com/login.aspx?direct=true&db=psyh&AN=2001-95022-290&site=ehost-live&scope=site NS  -

Kennedy, P. (2001). Working with survivors of torture in Sarajevo with Reiki. *Complement Ther Nurs Midwifery*, *7*(1 PG-4–7), 4–7. https://doi.org/10.1054/ctnm.2000.0516

Kerr, T., Walsh, J., & Marshall, A. (2001). Emotional change processes in music-assisted reframing. *J Music Ther*, *38*(3 PG-193–211), 193–211. https://doi.org/10.1093/jmt/38.3.193

Khazaei, D., Setarehdan, S. K., & Mehran, Y. Z. (2016). The effectiveness of music on human biological signals. *Biomedical Engineering - Applications, Basis and Communications*, *28*(1 PG-). https://doi.org/10.4015/S1016237216500022

Kirk, A. E. (2015). *Dance/movement therapy for adult women with posttraumatic stress disorder: A quasi-experimental study of symptom reduction and integration*. ProQuest Information & Learning. Retrieved from http://search.ebscohost.com/login.aspx?direct=true&db=psyh&AN=2015-99110-119&site=ehost-live&scope=site NS  -

Koch, S. C., Morlinghaus, K., & Fuchs, T. (2007). The joy dance. Specific effects of a single dance intervention on psychiatric patients with depression. *Arts in Psychotherapy*, *34*(4 PG-340–349), 340–349. https://doi.org/10.1016/j.aip.2007.07.001

Koch, S. C., Riege, R. F. F., Tisborn, K., Biondo, J., Martin, L., & Beelmann, A. (2019). Effects of Dance Movement Therapy and Dance on Health-Related Psychological Outcomes. A Meta-Analysis Update. *Front Psychol*, *10*(PG-1806), 1806. https://doi.org/10.3389/fpsyg.2019.01806

Koch, S. C., Wirtz, G., Harter, C., Weisbrod, M., Winkler, F., Pröger, A., & Herpertz, S. C. (2019). Embodied Self in Trauma and Self-Harm: A Pilot Study of Effects of Flamenco Therapy on Traumatized Inpatients. *Journal of Loss & Trauma*, *24*(5/6 PG-441–459), 441–459. https://doi.org/10.1080/15325024.2018.1507472

Körlin, D. (2008). Music breathing: Breath grounding and modulation of the Bonny Method of Guided Imagery and Music (BMGIM): Theory, method, and consecutive cases. *Journal of the Association for Music & Imagery*, *11*(PG-79-113), 79–113. Retrieved from http://search.ebscohost.com/login.aspx?direct=true&db=psyh&AN=2015-06293-005&site=ehost-live&scope=site dag.korlin@telia.com NS  -

Körlin, D., Nybäck, H., & Goldberg, F. S. (2000). Creative arts groups in psychiatric care: Development and evaluation of a therapeutic alternative. *Nordic Journal of Psychiatry*, *54*(5 PG-333–340), 333–340. https://doi.org/10.1080/080394800457165

Kosugi, N., Oshiyama, C., Kodama, N., & Niwa, S.-I. (2019). Incorporating music therapy into cognitive remediation to improve both cognitive dysfunction and negative symptoms in schizophrenia. *Schizophrenia Research*. Elsevier B.V. https://doi.org/10.1016/j.schres.2018.08.012

Kronsted, C. (2018). The self and dance movement therapy – a narrative approach. *Phenomenology and the Cognitive Sciences*, (PG-). https://doi.org/10.1007/s11097-018-9602-y

Kumar Dr, G., & Singh, B. K. (2013). To access the role of music therapy in depression and their comparison with drug therapy. *International Journal of Pharmaceutical Sciences and Research*, *4*(8 PG-3099–3102), 3099–3102. https://doi.org/10.13040/IJPSR.0975-8232

Kwan, C. K., & Clift, S. (2018). Exploring the processes of change facilitated by musical activities on mental wellness. *Nordic Journal of Music Therapy*, *27*(2 PG-142–157), 142–157. https://doi.org/10.1080/08098131.2017.1363808

Kwon, M., Gang, M., & Oh, K. (2013). Effect of the Group Music Therapy on Brain Wave, Behavior, and Cognitive Function among Patients with Chronic Schizophrenia. *Asian Nurs Res (Korean Soc Nurs Sci)*, *7*(4 PG-168–74), 168–174. https://doi.org/10.1016/j.anr.2013.09.005

Lai, Y. M. (1999). Effects of music listening on depressed women in Taiwan. *Issues Ment Health Nurs*, *20*(3 PG-229–46), 229–246. https://doi.org/10.1080/016128499248637

Landis-Shack, N., Heinz, A. J., & Bonn-Miller, M. O. (2017). Music Therapy for Posttraumatic Stress in Adults: A Theoretical Review. *Psychomusicology*, *27*(4 PG-334–342), 334–342. https://doi.org/10.1037/pmu0000192

Langdon, G. S., Pearson, J., Stastny, P., & Thorning, H. (1989). The integration of music therapy into a treatment approach in the transition of adult psychiatric patients from institution to community. *Music Therapy*, *8*(1 PG-92–107), 92–107. https://doi.org/10.1093/mt/8.1.92

Langston, J. M. (2019). *The lived experiences of adult male trauma survivors with dance movement therapy*. ProQuest Information & Learning. Retrieved from http://search.ebscohost.com/login.aspx?direct=true&db=psyh&AN=2019-41135-092&site=ehost-live&scope=site NS  -

Lantos, J., & Davies, A. L. (2000). Program description for the exposure to violence program. *Journal of Community Psychology*, *28*(6 PG-683–686), 683–686. https://doi.org/10.1002/1520-6629(200011)28:6<683::AID-JCOP10>3.0.CO;2-A

Leavitt, V. M., & Tunick, P. A. (2017). Friday music with Paul. *JAMA Neurology*, *74*(11 PG-1293), 1293. https://doi.org/10.1001/jamaneurol.2017.3009

Lee, H. J., Jang, S. H., Lee, S. Y., & Hwang, K. S. (2015). Effectiveness of dance/movement therapy on affect and psychotic symptoms in patients with schizophrenia. *Arts in Psychotherapy*, *45*(PG-64-68), 64–68. https://doi.org/10.1016/j.aip.2015.07.003

Lee, T. C. (2014). Trilogy of body imaginary: Dance/movement therapy for a psychiatric patient with depression. *Arts in Psychotherapy*, *41*(4 PG-400–408), 400–408. https://doi.org/10.1016/j.aip.2014.07.006

Lee, T. C., Lin, Y. S., Chiang, C. H., & Wu, M. H. (2013). Dance/movement therapy for children suffering from earthquake trauma in Taiwan: A preliminary exploration. *Arts in Psychotherapy*, *40*(1 PG-151–157), 151–157. https://doi.org/10.1016/j.aip.2012.12.002

Lee, W. J., Choi, S. H., Shin, J. E., Oh, C. Y., Ha, N. H., Lee, U. S., … Kang, D. H. (2018). Effects of an Online Imagery-Based Treatment Program in Patients with Workplace-Related Posttraumatic Stress Disorder: A Pilot Study. *Psychiatry Investig*, *15*(11 PG-1071–1078), 1071–1078. https://doi.org/10.30773/pi.2018.09.28

Lehrer-carle, I. (1971). Group dynamics as applied to the use of music with schizophrenic adolescents. *Journal of Contemporary Psychotherapy: On the Cutting Edge of Modern Developments in Psychotherapy*, *3*(2 PG-111–116), 111–116. https://doi.org/10.1007/BF02110244

LeLieuvre, R. B. (1998). “Goodnight Saigon”: Music, fiction, poetry, and film in readjustment group counseling. *Professional Psychology: Research and Practice*, *29*(1 PG-74–78), 74–78. https://doi.org/10.1037/0735-7028.29.1.74

Leubner, D., & Hinterberger, T. (2017). Reviewing the Effectiveness of Music Interventions in Treating Depression. *Front Psychol*, *8*(PG-1109), 1109. https://doi.org/10.3389/fpsyg.2017.01109

Leung, C. M., Lee, G., Cheung, B., Kwong, E., Wing, Y. K., Kan, C. S., & Lau, J. (1998). Karaoke therapy in the rehabilitation of mental patients. *Singapore Med J*, *39*(4 PG-166–8), 166–168. Retrieved from NS  -

Levine, B., & Land, H. M. (2016). A Meta-Synthesis of Qualitative Findings About Dance/Movement Therapy for Individuals With Trauma. *Qual Health Res*, *26*(3 PG-330–44), 330–344. https://doi.org/10.1177/1049732315589920

Levine, B. (2015). *Developing a body-oriented intervention for trauma*. ProQuest Information & Learning. Retrieved from http://search.ebscohost.com/login.aspx?direct=true&db=psyh&AN=2015-99090-192&site=ehost-live&scope=site NS  -

Lightstone, A. J., Bailey, S. K., & Voros, P. (2015). Collaborative music therapy via remote video technology to reduce a veteran’s symptoms of severe, chronic PTSD. *Arts & Health: International Journal for Research, Policy & Practice*, *7*(2 PG-123–136), 123–136. https://doi.org/10.1080/17533015.2015.1019895

Lin, M. F., Hsu, M. C., Chang, H. J., Hsu, Y. Y., Chou, M. H., & Crawford, P. (2010). Pivotal moments and changes in the Bonny Method of Guided Imagery and Music for patients with depression. *J Clin Nurs*, *19*(7-8 PG-1139–48), 1139–1148. https://doi.org/10.1111/j.1365-2702.2009.03140.x

Lipe, A. W., Ward, K. C., Watson, A. T., Manley, K., Keen, R., Kelly, J., & Clemmer, J. (2012). The effects of an arts intervention program in a community mental health setting: A collaborative approach. *Arts in Psychotherapy*, *39*(1 PG-25–30), 25–30. https://doi.org/10.1016/j.aip.2011.11.002

Lippi, S., & Petit, L. (2017). Subverting Space: An Exploration of a Dance Therapy Workshop Apparatus for Schizophrenics. *Psychoanal Rev*, *104*(2 PG-231–252), 231–252. https://doi.org/10.1521/prev.2017.104.2.231

Litchke, L., & Finley, C. (2019). Social-Emotional Benefits of Drumtastic Ability Beats® Dyadic Partnership between a College Veteran with PTSD and an Elementary Student in a Special Education Setting. *Therapeutic Recreation Journal*, *53*(2 PG-175–184), 175–184. https://doi.org/10.18666/TRJ-2019-V53-I2-9129

Longacre, M., Silver-Highfield, E., Lama, P., & Grodin, M. (2012). Complementary and alternative medicine in the treatment of refugees and survivors of torture: a review and proposal for action. *Torture*, *22*(1 PG-38–57), 38–57. Retrieved from NS  -

Lotter, C., & van Staden, W. (2019). Verbal affordances of active and receptive music therapy methods in major depressive disorder and schizophrenia-spectrum disorder. *Arts in Psychotherapy*, *64*(PG-59-68), 59–68. https://doi.org/10.1016/j.aip.2018.12.002

Lu, S. F., Lo, C. H., Sung, H. C., Hsieh, T. C., Yu, S. C., & Chang, S. C. (2013). Effects of group music intervention on psychiatric symptoms and depression in patient with schizophrenia. *Complement Ther Med*, *21*(6 PG-682–8), 682–688. https://doi.org/10.1016/j.ctim.2013.09.002

Lucas, D., & Ludwik, R. G. (1964). GROUP PSYCHOTHERAPY WITH DEPRESSED PATIENTS INCORPORATING “MOOD” MUSIC. *Am J Psychother*, *18*(PG-126-37), 126–137. https://doi.org/10.1176/appi.psychotherapy.1964.18.1.126

Luna, R. M. (2019). *An examination of a sound healing intervention as an adjunct to psychotherapy for depression*. ProQuest Information & Learning. Retrieved from http://search.ebscohost.com/login.aspx?direct=true&db=psyh&AN=2018-52510-041&site=ehost-live&scope=site NS  -

Lutgens, D., Gariepy, G., & Malla, A. (2017). Psychological and psychosocial interventions for negative symptoms in psychosis: systematic review and meta-analysis. *Br J Psychiatry*, *210*(5 PG-324–332), 324–332. https://doi.org/10.1192/bjp.bp.116.197103

Macfarlane, C., Masthoff, E., & Hakvoort, L. (2019). Short-Term Music Therapy Attention and Arousal Regulation Treatment (SMAART) for Prisoners with Posttraumatic Stress Disorder: A Feasibility Study. *Journal of Forensic Psychology Research and Practice*, *19*(5 PG-376–392), 376–392. https://doi.org/10.1080/24732850.2019.1670023

Magnis, E. S. (2013). *Finding a vocal path through depression*. ProQuest Information & Learning. Retrieved from http://search.ebscohost.com/login.aspx?direct=true&db=psyh&AN=2013-99120-228&site=ehost-live&scope=site NS  -

Mala, A., Karkou, V., & Meekums, B. (2012). Dance/Movement Therapy (D/MT) for depression: A scoping review. *Arts in Psychotherapy*, *39*(4 PG-287–295), 287–295. https://doi.org/10.1016/j.aip.2012.04.002

Maratos, A. S., Gold, C., Wang, X., & Crawford, M. J. (2008). Music therapy for depression. *Cochrane Database of Systematic Reviews*. John Wiley and Sons Ltd. https://doi.org/10.1002/14651858.CD004517.pub2

Margariti, A., Ktonas, P., Hondraki, P., Daskalopoulou, E., Kyriakopoulos, G., Economou, N. T., … Vaslamatzis, G. (2012). An application of the Primitive Expression form of dance therapy in a psychiatric population. *Arts in Psychotherapy*, *39*(2 PG-95–101), 95–101. https://doi.org/10.1016/j.aip.2012.01.001

Martin, D. W., & Beaver, N. (1951). A preliminary report on the use of the dance as an adjuvant in the therapy of schizophrenics. *Psychiatr Q Suppl*, *25*(2 PG-176–90), 176–190. Retrieved from NS  -

McCaffrey, T., Edwards, J., & Fannon, D. (2011). Is there a role for music therapy in the recovery approach in mental health? *Arts in Psychotherapy*, *38*(3 PG-185–189), 185–189. https://doi.org/10.1016/j.aip.2011.04.006

McCollum, J. D. (2001). *Audioscriptotherapy as an adjunct to psychotherapy for adult female survivors of childhood sexual abuse*. ProQuest Information & Learning. Retrieved from http://search.ebscohost.com/login.aspx?direct=true&db=psyh&AN=2001-95020-184&site=ehost-live&scope=site NS  -

McDonald, J., Codrea, A., & Gavin, B. (2015). A pilot music group for young people attending a community Child and Adolescent Mental Health Service. *Ir J Psychol Med*, *32*(3 PG-259–264), 259–264. https://doi.org/10.1017/ipm.2014.84

McGee, T. F., Williams, M., Racusen, F. R., & Cowen, J. (1968). Further evaluation of small group living program with schizophrenics. *Arch Gen Psychiatry*, *19*(6 PG-717–26), 717–726. https://doi.org/10.1001/archpsyc.1968.01740120077011

McKinney, C. H., & Grocke, D. E. (2016). The Bonny Method of Guided Imagery and Music for medical populations: Evidence for effectiveness and vision for the future. *Music and Medicine*, *8*(2 PG-18–25), 18–25. Retrieved from http://search.ebscohost.com/login.aspx?direct=true&db=psyh&AN=2016-22475-004&site=ehost-live&scope=site mckinneych@appstate.edu NS  -

Meekums, B., Karkou, V., & Nelson, E. A. (2015). Dance movement therapy for depression. *Cochrane Database Syst Rev*, (2 PG-Cd009895), Cd009895. https://doi.org/10.1002/14651858.CD009895.pub2

Metzger, L. K. (1991). A study of the musical preference of psychiatric patients in a short-term treatment center. *The Arts in Psychotherapy*, *18*(4 PG-357–358), 357–358. https://doi.org/10.1016/0197-4556(91)90076-M

Metzner, S., Verhey, J., Braak, P., & Hots, J. (2018). Auditory sensitivity in survivors of torture, political violence and flight—An exploratory study on risks and opportunities of music therapy. *Arts in Psychotherapy*, *58*(PG-33-41), 33–41. https://doi.org/10.1016/j.aip.2018.02.001

Metzner, S., Jaeger, U., Masuhr, O., Olschewski, U., Gräfe, E., Böske, A. C., & Dümpelmann, M. (2018). Forms of attunement during the initial stages of music therapy for patients with acute psychosis - A multicentre clinical study. *Nordic Journal of Music Therapy*, *27*(5 PG-360–380), 360–380. https://doi.org/10.1080/08098131.2018.1478879

Mezey, G., Durkin, C., & Krljes, S. (2015). Finding a voice – the feasibility and impact of setting up a community choir in a forensic secure setting. *Journal of Forensic Psychiatry and Psychology*, *26*(6 PG-781–797), 781–797. https://doi.org/10.1080/14789949.2015.1069881

Mitchell, S. D., & Zanker, A. (1949). Musical styles and mental disorders. *Occup. Ther. Rehab. Baltim.*, *28*(5 PG-411–422), 411–422. Retrieved from http://www.embase.com/search/results?subaction=viewrecord&from=export&id=L280674541 NS  -

Mitchell, S. D., & Zanker, A. (1948). The use of music in group therapy. *Journal of Mental Science*, *94*(397 PG-56–59), 56–59. Retrieved from http://www.embase.com/search/results?subaction=viewrecord&from=export&id=L280588966 NS  -

Mitchell, S. D. (1948). Music in mental hospitals. *Hospital, London*, *44*(PG-431-436), 431–436. Retrieved from http://search.ebscohost.com/login.aspx?direct=true&db=psyh&AN=1949-06266-001&site=ehost-live&scope=site NS  -

Moe, T., Roesen, A., & Raben, H. (2000). Restitutional factors in group music therapy with psychiatric patients based on a modification of guided imagery and music (GIM). *Nordic Journal of Music Therapy*, *9*(2 PG-36–50), 36–50. Retrieved from http://search.ebscohost.com/login.aspx?direct=true&db=rzh&AN=107042110&site=ehost-live&scope=site NS  -

Moe, T. (2002). Restitutional factors in receptive group music therapy inspired by GIM: The relationship between self-objects, psychological defence maneouvres and restitutional factors: Towards a theory. *Nordic Journal of Music Therapy*, *11*(2 PG-152–166), 152–166. https://doi.org/10.1080/08098130209478057

Mohammadi, A. Z., Minhas, L. S., Haidari, M., & Panah, F. M. (2012). A study of the effects of music therapy on negative and positive symptoms in schizophrenic patients. *German Journal of Psychiatry*, *15*(2 PG-56–62), 56–62. Retrieved from http://www.embase.com/search/results?subaction=viewrecord&from=export&id=L365334969 http://www.gjpsy.uni-goettingen.de/gjp-article-mohammadi.pdf NS  -

Morgan, K. A. (2018). *Music therapy in the management of acute psychosis*. ProQuest Information & Learning. Retrieved from http://search.ebscohost.com/login.aspx?direct=true&db=psyh&AN=2017-51411-139&site=ehost-live&scope=site NS  -

Mossler, K., Chen, X., Heldal, T. O., & Gold, C. (2011). Music therapy for people with schizophrenia and schizophrenia-like disorders. *Cochrane Database Syst Rev*, (12 PG-Cd004025), Cd004025. https://doi.org/10.1002/14651858.CD004025.pub3

Mu, Z., Chang, Y., Xu, J., Pang, X., Zhang, H., Liu, X., … Wan, Y. (2016). Pre-attentive dysfunction of musical processing in major depressive disorder: A mismatch negativity study. *J Affect Disord*, *194*(PG-50-6), 50–56. https://doi.org/10.1016/j.jad.2016.01.028

Muller, W., Haffelder, G., Schlotmann, A., Schaefers, A. T., & Teuchert-Noodt, G. (2014). Amelioration of psychiatric symptoms through exposure to music individually adapted to brain rhythm disorders - a randomised clinical trial on the basis of fundamental research. *Cogn Neuropsychiatry*, *19*(5 PG-399–413), 399–413. https://doi.org/10.1080/13546805.2013.879054

Negreiros-Vianna, M., & Costa, C. M. (2011). Action / Relationship / Communication: A Music Therapy Method for Schizophrenia. *Voices: A World Forum for Music Therapy*, *11*(3 PG-np), np. Retrieved from https://search.proquest.com/docview/1370898215?accountid=11752 http://findit.library.jhu.edu/resolve?url_ver=Z39.88-2004&rft_val_fmt=info:ofi/fmt:kev:mtx:journal&genre=article&sid=ProQ:ProQ%3Aiimpft&atitle=Action+%2F+Relationship+%2F+Communication%3A+A+Mu

Norr, A. M. (2018). *Amelioration of anxiety sensitivity cognitive concerns: Exposure to dissociative symptoms*. ProQuest Information & Learning. Retrieved from http://search.ebscohost.com/login.aspx?direct=true&db=psyh&AN=2018-11222-008&site=ehost-live&scope=site NS  -

Noyes, E. M., & Schlesinger, J. J. (2017). ICU-related PTSD – A review of PTSD and the potential effects of collaborative songwriting therapy. *Journal of Critical Care*, *42*(PG-78-84), 78–84. https://doi.org/10.1016/j.jcrc.2017.06.014

Nucci, A. (1978). *The use of music in individual psychotherapy*. ProQuest Information & Learning. Retrieved from http://search.ebscohost.com/login.aspx?direct=true&db=psyh&AN=1979-11288-001&site=ehost-live&scope=site NS  -

Nunez, S. (2006). *Effects of drumming on anxiety in Latino male youth*. ProQuest Information & Learning. Retrieved from http://search.ebscohost.com/login.aspx?direct=true&db=psyh&AN=2006-99014-193&site=ehost-live&scope=site NS  -

Odell-Miller, H., Hughes, P., & Westacott, M. (2006). An investigation into the effectiveness of the arts therapies for adults with continuing mental health problems. *Psychotherapy Research*, *16*(1 PG-122–139), 122–139. https://doi.org/10.1080/10503300500268342

Odell, H. (1988). A music therapy approach in mental health. *Psychology of Music*, *16*(1 PG-52–61), 52–61. https://doi.org/10.1177/0305735688161005

Offer, D., & Stine, D. (1960). Function of music in spontaneous art productions. *Archives of General Psychiatry*, *3*(5 PG-490–503), 490–503. Retrieved from http://www.embase.com/search/results?subaction=viewrecord&from=export&id=L281061659 NS  -

Oganesian, N. (2008). Dance therapy as form of communication activating psychotherapy for schizophrenic patients. *Body, Movement and Dance in Psychotherapy*, *3*(2 PG-97–106), 97–106. https://doi.org/10.1080/17432970802080057

Orjasaeter, K. B., Davidson, L., Hedlund, M., Bjerkeset, O., & Ness, O. (2018). “I now have a life!” Lived experiences of participation in music and theater in a mental health hospital. *PLoS One*, *13*(12 PG-e0209242), e0209242. https://doi.org/10.1371/journal.pone.0209242

Orjasaeter, K. B., & Ness, O. (2017). Acting Out: Enabling Meaningful Participation Among People With Long-Term Mental Health Problems in a Music and Theater Workshop. *Qual Health Res*, *27*(11 PG-1600–1613), 1600–1613. https://doi.org/10.1177/1049732316679954

Orth, J. (2005). Music Therapy with Traumatized Refugees in a Clinical Setting. *Voices: A World Forum for Music Therapy*, *5*(2 PG-np), np. Retrieved from https://search.proquest.com/docview/1367899413?accountid=11752 http://findit.library.jhu.edu/resolve?url_ver=Z39.88-2004&rft_val_fmt=info:ofi/fmt:kev:mtx:journal&genre=article&sid=ProQ:ProQ%3Aiimpft&atitle=Music+Therapy+with+Traumatized+Refugees+in+a+Clin

Osborne, N. (2012). Neuroscience and “real world” practice: music as a therapeutic resource for children in zones of conflict. *Ann N Y Acad Sci*, *1252*(PG-69-76), 69–76. https://doi.org/10.1111/j.1749-6632.2012.06473.x

Osuch, E. A., Bluhm, R. L., Williamson, P. C., Theberge, J., Densmore, M., & Neufeld, R. W. (2009). Brain activation to favorite music in healthy controls and depressed patients. *Neuroreport*, *20*(13 PG-1204–8), 1204–1208. https://doi.org/10.1097/WNR.0b013e32832f4da3

Papadopoulos, N. L. R., & Röhricht, F. (2014). An investigation into the application and processes of manualised group body psychotherapy for depressive disorder in a clinical trial. *Body, Movement & Dance in Psychotherapy*, *9*(3 PG-167–180), 167–180. https://doi.org/10.1080/17432979.2013.847499

Parquet, S. C. (2017). Effects of Music Therapy on Depressed Adolescents. *Effects of Music Therapy on Depressed Adolescents*, (PG-1-1), 1. Retrieved from http://search.ebscohost.com/login.aspx?direct=true&db=rzh&AN=129468754&site=ehost-live&scope=site NS  -

Parslow, R., Morgan, A. J., Allen, N. B., Jorm, A. F., O’Donnell, C. P., & Purcell, R. (2008). Effectiveness of complementary and self-help treatments for anxiety in children and adolescents. *Med J Aust*, *188*(6 PG-355–9), 355–359. Retrieved from https://onlinelibrary.wiley.com/doi/full/10.5694/j.1326-5377.2008.tb01654.x?sid=nlm%3Apubmed NS  -

Pavlicevic, M., & Trevarthen, C. (1989). A musical assessment of psychiatric states in adults. *Psychopathology*, *22*(6 PG-325–34), 325–334. https://doi.org/10.1159/000284615

Pavlicevic, M., Trevarthen, C., & Duncan, J. (1994). Improvisational Music-Therapy and the Rehabilitation of Persons Suffering From Chronic-Schizophrenia. *Journal of Music Therapy*, *31*(PG-86‐104), 86‐104. Retrieved from https://www.cochranelibrary.com/central/doi/10.1002/central/CN-00660124/full NS  -

Pavlov, A., Kameg, K., Cline, T. W., Chiapetta, L., Stark, S., & Mitchell, A. M. (2017). Music Therapy as a Nonpharmacological Intervention for Anxiety in Patients with a Thought Disorder. *Issues Ment Health Nurs*, *38*(3 PG-285–288), 285–288. https://doi.org/10.1080/01612840.2016.1264516

Payn, S. B. (1974). Musical associations in psychotherapy. *Am J Psychother*, *28*(2 PG-288–91), 288–291. https://doi.org/10.1176/appi.psychotherapy.1974.28.2.288

Pedersen, I. N., Bonde, L. O., Hannibal, N. J., Nielsen, J., Aagaard, J., Bertelsen, L. R., … Nielsen, R. E. (2019). Music Therapy as Treatment of Negative Symptoms for Adult Patients Diagnosed with Schizophrenia-Study Protocol for a Randomized, Controlled and Blinded Study. *Medicines (Basel)*, *6*(2 PG-). https://doi.org/10.3390/medicines6020046

Peng, S. M., Koo, M., & Kuo, J. C. (2010). Effect of group music activity as an adjunctive therapy on psychotic symptoms in patients with acute schizophrenia. *Arch Psychiatr Nurs*, *24*(6 PG-429–34), 429–434. https://doi.org/10.1016/j.apnu.2010.04.001

Petrovsky, D., Cacchione, P. Z., & George, M. (2015). Review of the effect of music interventions on symptoms of anxiety and depression in older adults with mild dementia. *Int Psychogeriatr*, *27*(10 PG-1661–70), 1661–1670. https://doi.org/10.1017/s1041610215000393

Pezzin, L. E., Larson, E. R., Lorber, W., McGinley, E. L., & Dillingham, T. R. (2018). Music-instruction intervention for treatment of post-traumatic stress disorder: a randomized pilot study. *BMC Psychol*, *6*(1 PG-60), 60. https://doi.org/10.1186/s40359-018-0274-8

Pinniger, R., Brown, R. F., Thorsteinsson, E. B., & McKinley, P. (2012). Argentine tango dance compared to mindfulness meditation and a waiting-list control: a randomised trial for treating depression. *Complement Ther Med*, *20*(6 PG-377–84), 377–384. https://doi.org/10.1016/j.ctim.2012.07.003

Pixler, L. (2017). Psychedelic movement: Healing trauma through MDMA (3,4- methylenedioxymethamphetamine)-assisted authentic movement psychotherapy. *Journal of Transpersonal Psychology*, *49*(2 PG-121–135), 121–135. Retrieved from http://search.ebscohost.com/login.aspx?direct=true&db=psyh&AN=2018-63645-004&site=ehost-live&scope=site lyndsaypixler@gmail.com NS  -

Porter, S., Holmes, V., McLaughlin, K., Lynn, F., Cardwell, C., Braiden, H. J., … Rogan, S. (2012). Music in mind, a randomized controlled trial of music therapy for young people with behavioural and emotional problems: study protocol. *J Adv Nurs*, *68*(10 PG-2349–58), 2349–2358. https://doi.org/10.1111/j.1365-2648.2011.05936.x

Precin, P. (2011). Occupation as therapy for trauma recovery: a case study. *Work*, *38*(1 PG-77–81), 77–81. https://doi.org/10.3233/wor-2011-1106

Preyde, M., Berends, A., Parehk, S., & Heintzman, J. (2017). Adolescents’ Evaluation of Music Therapy in an Inpatient Psychiatric Unit: A Quality Improvement Project. *Music Therapy Perspectives*, *35*(1 PG-58–62), 58–62. https://doi.org/10.1093/mtp/miv008

Priebe, S., Savill, M., Wykes, T., Bentall, R., Lauber, C., Reininghaus, U., … Rohricht, F. (2016). Clinical effectiveness and cost-effectiveness of body psychotherapy in the treatment of negative symptoms of schizophrenia: a multicentre randomised controlled trial. *Health Technol Assess*, *20*(11 PG-vii–xxiii, 1–100), vii–xxiii, 1. https://doi.org/10.3310/hta20110

Punkanen, M., Eerola, T., & Erkkila, J. (2011). Biased emotional recognition in depression: perception of emotions in music by depressed patients. *J Affect Disord*, *130*(1-2 PG-118–26), 118–126. https://doi.org/10.1016/j.jad.2010.10.034

Punkanen, M., Saarikallio, S., & Luck, G. (2014). Emotions in motion: Short-term group form Dance/Movement Therapy in the treatment of depression: A pilot study. *Arts in Psychotherapy*, *41*(5 PG-493–497), 493–497. https://doi.org/10.1016/j.aip.2014.07.001

Pylvanainen, P. M., Muotka, J. S., & Lappalainen, R. (2015). A dance movement therapy group for depressed adult patients in a psychiatric outpatient clinic: effects of the treatment. *Front Psychol*, *6*(PG-980), 980. https://doi.org/10.3389/fpsyg.2015.00980

Pylvänäinen, P., & Lappalainen, R. (2018). Change in body image among depressed adult outpatients after a dance movement therapy group treatment. *Arts in Psychotherapy*, *59*(PG-34-45), 34–45. https://doi.org/10.1016/j.aip.2017.10.006

Rafique, R., Anjum, A., & Raheem, S. S. (2019). Efficacy of Surah Al-Rehman in Managing Depression in Muslim Women. *J Relig Health*, *58*(2 PG-516–526), 516–526. https://doi.org/10.1007/s10943-017-0492-z

Ragg, D. M., Soulliere, J., & Turner, M. (2019). Drumming and Mindfulness Integrations into an Evidence-Based Group Intervention. *Social Work with Groups*, *42*(1 PG-29–42), 29–42. https://doi.org/10.1080/01609513.2017.1402401

Ramaswamy, A. (2014). Natya yoga therapy: Using movement and music to create meditative relief in schizophrenia (based on ashta anga yoga). *Action Research*, *12*(3 PG-237–253), 237–253. https://doi.org/10.1177/1476750314534454

Ready, T. (2012). *Music as container*. ProQuest Information & Learning. Retrieved from http://search.ebscohost.com/login.aspx?direct=true&db=psyh&AN=2012-99180-307&site=ehost-live&scope=site NS  -

Reeves, P. D. (2014). *How music and lyrics protect and heal the souls of african american women who have experienced domestic-violence trauma, sexual abuse, or depression: A phenomenological study*. ProQuest Information & Learning. Retrieved from http://search.ebscohost.com/login.aspx?direct=true&db=psyh&AN=2014-99020-144&site=ehost-live&scope=site NS  -

Reschke-Hernandez, A. E. (2014). Paula Lind Ayers: “song-physician” for troops with shell shock during World War I. *J Music Ther*, *51*(3 PG-276–91), 276–291. https://doi.org/10.1093/jmt/thu022

Robarts, J. (2006). Music therapy with sexually abused children. *Clinical Child Psychology & Psychiatry*, *11*(2 PG-249–269), 249–269. Retrieved from http://search.ebscohost.com/login.aspx?direct=true&db=rzh&AN=106326023&site=ehost-live&scope=site NS  -

Robertson-Gillam, K. (2018). *Reducing major depression in mid to later life with a choir therapy program: A mixed methods study*. ProQuest Information & Learning. Retrieved from http://search.ebscohost.com/login.aspx?direct=true&db=psyh&AN=2017-51411-283&site=ehost-live&scope=site NS  -

Romero, E. F., Hurwitz, A. J., & Carranza, V. (1983). Dance therapy on a therapeutic community for schizophrenic patients. *Arts in Psychotherapy*, *10*(2 PG-85–92), 85–92. https://doi.org/10.1016/0197-4556(83)90034-5

Rubin, B. (1973). Music therapy in an outreach station of the Milwaukee County Mental Health Center. *J.MUSIC THER.*, *10*(4 PG-201–204), 201–204. Retrieved from http://www.embase.com/search/results?subaction=viewrecord&from=export&id=L4144480 NS  -

Rubin, H. E., & Katz, E. (1946). Auroratone films for the treatment of psychotic depressions in an Army general hospital. *Journal of Clinical Psychology*, *2*(PG-333-340), 333–340. https://doi.org/10.1002/1097-4679(194610)2:4<333::AID-JCLP2270020405>3.0.CO;2-P

Rudstam, G., Elofsson, U., Søndergaard, H. P., Bonde, L. O., & Beck, B. D. (2017). Trauma-focused group music and imagery with women suffering from PTSD/complex PTSD: A feasibility study. *Collected Work: Approaches: An Interdisciplinary Journal of Music Therapy/Approaches: Ena Diepistīmoniko Periodiko Mousikotherapeias. IX/2 (2017): Guided Imagery and Music: Contemporary European Perspectives and Developments. Published by: Ellīnikou Syllo*, *9*(2 PG-208–218), 208–218. Retrieved from http://search.ebscohost.com/login.aspx?direct=true&db=rih&AN=A1246688&site=ehost-live&scope=site http://approaches.gr/rudstam-a20171222/ NS  -

Russell, L. A. (1988). *Comparisons of cognitive, music and imagery techniques on anxiety reduction*. ProQuest Information & Learning. Retrieved from http://search.ebscohost.com/login.aspx?direct=true&db=psyh&AN=1988-57005-001&site=ehost-live&scope=site NS  -

Sandel, S. L. (1982). The process of individuation in dance-movement therapy with schizophrenic patients. *Arts in Psychotherapy*, *9*(1 PG-11–18), 11–18. https://doi.org/10.1016/0197-4556(82)90022-3

Sandler, H., Fendel, U., Peters, E., Rose, M., Bösel, R., & Klapp, B. F. (2017). Subjective experience of relaxation – induced by vibroacoustic stimulation by a Body Monochord or CD music – a randomised, controlled study in patients with psychosomatic disorders. *Nordic Journal of Music Therapy*, *26*(1 PG-79–98), 79–98. https://doi.org/10.1080/08098131.2015.1089312

Schapira, D. (2003). Last Sounds of the Shipwreck: Aspects of the Plurimodal Method in the Treatment of Psychosis. *Nordic Journal of Music Therapy*, *12*(2 PG-163–172), 163–172. https://doi.org/10.1080/08098130309478087

Schmidt, K., & Hayashi, N. (2002). Musical therapy for inpatients with chronic psychosis. *Focus on Alternative & Complementary Therapies*, *7*(4 PG-367–368), 367–368. Retrieved from http://search.ebscohost.com/login.aspx?direct=true&db=rzh&AN=106701895&site=ehost-live&scope=site NS  -

Scholer, M., Lemétayer, F., & Schiltz, L. (2016). Revealing the creative process in music psychotherapy by applying a test of creative thinking and clinical observational frames. *Nordic Journal of Music Therapy*, *25*(3 PG-229–247), 229–247. https://doi.org/10.1080/08098131.2015.1034757

Shagan, S., Shagan, D., Shagan, B., Fiszdon, J. M., Thime, W., Haber, L. C., … Choi, J. (2018). How Music Impacts Visual Attention Training in Schizophrenia: A Pilot Study. *J Nerv Ment Dis*, *206*(12 PG-968–970), 968–970. https://doi.org/10.1097/nmd.0000000000000911

Sharma, P. (2014). Making Song, Making Sanity: Recovery from Bipolar Disorder. *Canadian Journal of Music Therapy*, *20*(1 PG-65–84), 65–84. Retrieved from http://search.ebscohost.com/login.aspx?direct=true&db=rzh&AN=107816330&site=ehost-live&scope=site NS  -

Shatin, L. (1957). The influence of rhythmic drumbeat stimuli upon the pulse rate and general activity of long-term schizophrenics. *J Ment Sci*, *103*(430 PG-172–88), 172–188. https://doi.org/10.1192/bjp.103.430.172

Shatin, L., & Zimet, C. (1958). Influence of music upon verbal participation in group psychotherapy. *Diseases of the Nervous System*, *19*(2 PG-66–72), 66–72. Retrieved from http://www.embase.com/search/results?subaction=viewrecord&from=export&id=L280957628 NS  -

Shealy, C. N., Cady, R. K., & Cox, R. H. (1995). Pain, stress and depression: Psychoneurophysiology and therapy. *Stress Medicine*, *11*(2 PG-75–77), 75–77. Retrieved from http://www.embase.com/search/results?subaction=viewrecord&from=export&id=L25147537 NS  -

Sherwin, A. C. (1953). Reactions to music of autistic (schizophrenic) children. *Am J Psychiatry*, *109*(11 PG-823–31), 823–831. https://doi.org/10.1176/ajp.109.11.823

Shih, Y. N., Chen, C. S., Chiang, H. Y., & Liu, C. H. (2015). Influence of background music on work attention in clients with chronic schizophrenia. *Work*, *51*(1 PG-153–8), 153–158. https://doi.org/10.3233/wor-141846

Sigurdardottir, G. A., Nielsen, P. M., Ronager, J., & Wang, A. G. (2019). A pilot study on high amplitude low frequency-music impulse stimulation as an add-on treatment for depression. *Brain Behav*, *9*(10 PG-e01399), e01399. https://doi.org/10.1002/brb3.1399

Silverman, M. J. (2016). Effects of Educational Music Therapy on State Hope for Recovery in Acute Care Mental Health Inpatients: A Cluster-Randomized Effectiveness Study. *Front Psychol*, *7*(PG-1569), 1569. https://doi.org/10.3389/fpsyg.2016.01569

Silverman, M. J. (2014). Effects of a live educational music therapy intervention on acute psychiatric inpatients’ perceived social support and trust in the therapist: a four-group randomized effectiveness study. *J Music Ther*, *51*(3 PG-228–49), 228–249. https://doi.org/10.1093/jmt/thu011

Silverman, M. J. (2019). Music Therapy for Coping Self-Efficacy in an Acute Mental Health Setting: A Randomized Pilot Study. *Community Ment Health J*, *55*(4 PG-615–623), 615–623. https://doi.org/10.1007/s10597-018-0319-8

Silverman, M. J. (2010). Perceptions of music therapy interventions from inpatients with severe mental illness: A mixed-methods approach. *Arts in Psychotherapy*, *37*(3 PG-264–268), 264–268. https://doi.org/10.1016/j.aip.2010.05.002

Silverman, M. J. (2003). Contingency songwriting to reduce combativeness and non-cooperation in a client with schizophrenia: A case study. *Arts in Psychotherapy*, *30*(1 PG-25–33), 25–33. https://doi.org/10.1016/S0197-4556(02)00231-9

Silverman, M. J. (2016). Effects of educational music therapy on illness management knowledge and mood state in acute psychiatric inpatients: A randomized three group effectiveness study. *Nordic Journal of Music Therapy*, *25*(1 PG-57–75), 57–75. https://doi.org/10.1080/08098131.2015.1008559

Silverman, M. J. (2003). The influence of music on the symptoms of psychosis: A meta-analysis. *Journal of Music Therapy*, *40*(1), 27–40. https://doi.org/10.1093/jmt/40.1.27

Skelly, C. G., & Haslerud, G. M. (1952). Music and the general activity of apathetic schizophrenics. *J Abnorm Psychol*, *47*(2 PG-188–92), 188–192. https://doi.org/10.1037/h0058664

Skrzypek, H. A. (2017). Body Movement Music Score—Introduction of a newly developed model for the analysis and description of body qualities, movement and music in music therapy. *Health Psychology Report*, *5*(2 PG-100–124), 100–124. https://doi.org/10.5114/hpr.2017.64628

Solli, H. P. (2008). “Shut up and play!”: Improvisational use of popular music for a man with schizophrenia. *Nordic Journal of Music Therapy*, *17*(1 PG-67–77), 67–77. Retrieved from http://search.ebscohost.com/login.aspx?direct=true&db=rzh&AN=105897599&site=ehost-live&scope=site NS  -

Solli, H. P., & Rolvsjord, R. (2015). “The Opposite of Treatment”: A qualitative study of how patients diagnosed with psychosis experience music therapy. *Nord J Music Ther*, *24*(1 PG-67–92), 67–92. https://doi.org/10.1080/08098131.2014.890639

Solli, H. P. (2014). *The groove of recovery: A qualitative study of how people diagnosed with psychosis experience music therapy*. Universitetet i Bergen/University of Bergen, Bergen. Retrieved from http://search.ebscohost.com/login.aspx?direct=true&db=rih&AN=A1146495&site=ehost-live&scope=site http://bora.uib.no/handle/1956/8753?show=full NS  -

Stanek, D. (2015). Bridging past and present: Embodied intergenerational trauma and the implications for dance/movement therapy. *Body, Movement and Dance in Psychotherapy*, *10*(2 PG-94–105), 94–105. https://doi.org/10.1080/17432979.2014.971872

Steinberg-Oren, S. L., Krasnova, M., Krasnov, I. S., Baker, M. R., & Ames, D. (2016). Let’s Dance: A Holistic Approach to Treating Veterans With Posttraumatic Stress Disorder. *Fed Pract*, *33*(7 PG-44–49), 44–49. Retrieved from https://www.ncbi.nlm.nih.gov/pmc/articles/PMC6366571/pdf/fp-33-07-44.pdf NS  -

Stephens, G. (1983). The use of improvisation for developing relatedness in the adult client. *Music Therapy*, *3*(1 PG-29–42), 29–42. https://doi.org/10.1093/mt/3.1.29

Stephens, G. (1981). Adele: A study in silence. *Music Therapy*, *1*(1 PG-25–31), 25–31. https://doi.org/10.1093/mt/1.1.25

Steward, K. (2018). All roads lead to where I stand: A veteran case review. *Music and Medicine*, *10*(3 PG-130–141), 130–141. Retrieved from http://search.ebscohost.com/login.aspx?direct=true&db=psyh&AN=2019-23902-002&site=ehost-live&scope=site pjkmstewart@optonline.net NS  -

Story, K. M., & Beck, B. D. (2017). Guided Imagery and Music with female military veterans: An intervention development study. *Arts in Psychotherapy*, *55*(PG-93-102), 93–102. https://doi.org/10.1016/j.aip.2017.05.003

Strauss, M., van Heerden, S. M., & Joubert, G. (2016). Occupational therapy and the use of music tempo in the treatment of the mental health care user with psychosis. *South African Journal of Occupational Therapy*, *46*(1 PG-21–26), 21–26. https://doi.org/10.17159/2310-3833/2016/v46n1a6

Sutton, J., & De Backer, J. (2009). Music, trauma and silence: The state of the art. *Arts in Psychotherapy*, *36*(2 PG-75–83), 75–83. https://doi.org/10.1016/j.aip.2009.01.009

Talwar, N., Crawford, M. J., Maratos, A., Nur, U., McDermott, O., & Procter, S. (2006). Music therapy for in-patients with schizophrenia: exploratory randomised controlled trial. *Br J Psychiatry*, *189*(PG-405-9), 405–409. https://doi.org/10.1192/bjp.bp.105.015073

Tan, S., Zou, Y., Wykes, T., Reeder, C., Zhu, X., Yang, F., … Zhou, D. (2016). Group cognitive remediation therapy for chronic schizophrenia: A randomized controlled trial. *Neurosci Lett*, *626*(PG-106-11), 106–111. https://doi.org/10.1016/j.neulet.2015.08.036

Tavormina, M. G. M., Tavormina, R., & Nemoianni, E. (2014). The singing-group: A new therapic rehabilitation for mood desorders. *Psychiatria Danubina*, *26*(PG-173-177), 173–177. Retrieved from http://www.embase.com/search/results?subaction=viewrecord&from=export&id=L600965502 NS  -

Tavormina, R., & Tavormina, M. G. M. (2017). Overcoming the Social Stigma on Mood Disorders with Dancing. *Psychiatr Danub*, *29*(Suppl 3 PG-427-431), 427–431. Retrieved from NS  -

Taylor, J. Y., & Holston, E. C. (2014). MAMBRA’s impact on IPV symptoms of incarcerated and formerly incarcerated women. *Issues Ment Health Nurs*, *35*(5 PG-344–55), 344–355. https://doi.org/10.3109/01612840.2013.868962

Thierry, H., Gunther-Cohen, N., Chamayou, C., Bonnet, A. M., Bloch, F., Wargon, I., & Le Ber, I. (2012). Neurofeedback in FTD and PSP patients: A new therapeutic tool in the treatment of emotion recognition? A proof-of-concept study. *Dementia and Geriatric Cognitive Disorders*, *34*(PG-23-24), 23–24. Retrieved from http://www.embase.com/search/results?subaction=viewrecord&from=export&id=L71371031 NS  -

Thomas, E. C., Snethen, G., O’Shea, A., Suarez, J., Hurford, I., & Salzer, M. S. (2019). An Examination of the Community Participation Interests of Young Adults with Serious Mental Illnesses. *J Behav Health Serv Res*, (PG-). https://doi.org/10.1007/s11414-019-09678-0

Tornek, A., Field, T., Hernandez-Reif, M., Diego, M., & Jones, N. (2003). Music effects on EEG in intrusive and withdrawn mothers with depressive symptoms. *Psychiatry*, *66*(3 PG-234–43), 234–243. https://doi.org/10.1521/psyc.66.3.234.25157

Traub, C. (1969). The relation of music to speech of low verbalizing subjects in a music listening activity. *Journal of Music Therapy*, *6*(4 PG-105–107), 105–107. https://doi.org/10.1093/jmt/6.4.105

Trimmer, C., Tyo, R., Pikard, J., McKenna, C., & Naeem, F. (2018). Low-Intensity Cognitive Behavioural Therapy-Based Music Group (CBT-Music) for the Treatment of Symptoms of Anxiety and Depression: A Feasibility Study. *Behav Cogn Psychother*, *46*(2 PG-168–181), 168–181. https://doi.org/10.1017/s1352465817000480

Tseng, P. T., Chen, Y. W., Lin, P. Y., Tu, K. Y., Wang, H. Y., Cheng, Y. S., … Wu, C. K. (2016). Significant treatment effect of adjunct music therapy to standard treatment on the positive, negative, and mood symptoms of schizophrenic patients: a meta-analysis. *BMC Psychiatry*, *16*(PG-16), 16. https://doi.org/10.1186/s12888-016-0718-8

Tyson, F. (1979). Child at the gate: Individual music therapy with a schizophrenic woman. *Arts in Psychotherapy*, *6*(2 PG-77–83), 77–83. Retrieved from http://www.embase.com/search/results?subaction=viewrecord&from=export&id=L9255573 NS  -

Tyson, F. (1987). Analytically-oriented music therapy in a case of generalized anxiety disorder. *Music Therapy Perspectives*, *4*(PG-51-55), 51–55. https://doi.org/10.1093/mtp/4.1.51

Ujike, S., Yasuhara, Y., Osaka, K., Sato, M., Catangui, E., Edo, S., … Mifune, K. (2019). Encounter of Pepper-CPGE for the elderly and patients with schizophrenia: an innovative strategy to improve patient’s recreation, rehabilitation, and communication. *J Med Invest*, *66*(1.2 PG-50–53), 50–53. https://doi.org/10.2152/jmi.66.50

Ulrich, G., Houtmans, T., & Gold, C. (2007). The additional therapeutic effect of group music therapy for schizophrenic patients: a randomized study. *Acta Psychiatr Scand*, *116*(5 PG-362–70), 362–370. https://doi.org/10.1111/j.1600-0447.2007.01073.x

van Westrhenen, N., & Fritz, E. (2014). Creative Arts Therapy as treatment for child trauma: An overview. *Arts in Psychotherapy*, *41*(5 PG-527–534), 527–534. https://doi.org/10.1016/j.aip.2014.10.004

Varga, E., Endre, S., Molnár, D., Tényi, T., & Herold, R. (2017). Efficacy of metacognitive training compared with a psychosocial rehabilitation program on social cognitive processing in schizophrenia. *European Neuropsychopharmacology*, *27*(PG-S959), S959. Retrieved from http://www.embase.com/search/results?subaction=viewrecord&from=export&id=L619297112 NS  -

Vasserman, D. S. (2019). *Thinking, moving, and feeling: A proposed movement and behavioral intervention for refugee children with trauma*. ProQuest Information & Learning. Retrieved from http://search.ebscohost.com/login.aspx?direct=true&db=psyh&AN=2019-41134-245&site=ehost-live&scope=site NS  -

Vaudreuil, R., Bronson, H., & Bradt, J. (2019). Bridging the Clinic to Community: Music Performance as Social Transformation for Military Service Members. *Front Psychol*, *10*(PG-119), 119. https://doi.org/10.3389/fpsyg.2019.00119

Veerman, S. R. T., Schulte, P. F. J., & de Haan, L. (2017). Treatment for Negative Symptoms in Schizophrenia: A Comprehensive Review. *Drugs*, *77*(13 PG-1423–1459), 1423–1459. https://doi.org/10.1007/s40265-017-0789-y

Ventouras, E. C., Margariti, A., Chondraki, P., Kalatzis, I., Economou, N. T., Tsekou, H., … Ktonas, P. (2015). EEG-based investigation of brain connectivity changes in psychotic patients undergoing the primitive expression form of dance therapy: a methodological pilot study. *Cogn Neurodyn*, *9*(2 PG-231–48), 231–248. https://doi.org/10.1007/s11571-014-9319-8

Volpe, U., Gianoglio, C., Autiero, L., Marino, M. L., Facchini, D., Mucci, A., & Galderisi, S. (2018). Acute Effects of Music Therapy in Subjects With Psychosis During Inpatient Treatment. *Psychiatry*, *81*(3 PG-218–227), 218–227. https://doi.org/10.1080/00332747.2018.1502559

von der Nahmer, M. (2019). Musikalisierung: How a Despondent Mind Shapes Thought into Music: A young boy’s journey &amp; music therapy from an insider’s point of view. *Music and Medicine (Online)*, *11*(1 PG-64), 64. Retrieved from https://search.proquest.com/docview/2194109149?accountid=11752 http://findit.library.jhu.edu/resolve?url_ver=Z39.88-2004&rft_val_fmt=info:ofi/fmt:kev:mtx:journal&genre=article&sid=ProQ:ProQ%3Aiimpft&atitle=Musikalisierung%3A+How+a+Despondent+Mind+Shapes+T

Wadeson, H. (1976). Combining expressive therapies. *American Journal of Art Therapy*, *15*(2 PG-43–46), 43–46. Retrieved from http://search.ebscohost.com/login.aspx?direct=true&db=psyh&AN=1976-21013-001&site=ehost-live&scope=site NS  -

Walker, J., & Boyce-Tillman, J. (2002). Music lessons on prescription? The impact of music lessons for children with chronic anxiety problems. *Health Education*, *102*(4 PG-172–179), 172–179. https://doi.org/10.1108/09654280210434246

Wang, S., & Agius, M. (2018). The use of music therapy in the treatment of mental illness and the enhancement of societal wellbeing. In *Psychiatria Danubina* (Vol. 30, pp. S595–S600). Medicinska Naklada Zagreb.

Weintraub, I. G. (1958). *An experimental investigation of the emotional reactions of schizophrenics to selected compositions of music*. Retrieved from http://search.ebscohost.com/login.aspx?direct=true&db=psyh&AN=1959-06700-001&site=ehost-live&scope=site NS  -

Wellman, R., & Pinkerton, J. (2015). The Development of a Music Therapy Protocol: A Music 4 Life Case Report of a Veteran with PTSD. *Music and Medicine (Online)*, *7*(3 PG-np), np. Retrieved from https://search.proquest.com/docview/1763719473?accountid=11752 http://findit.library.jhu.edu/resolve?url_ver=Z39.88-2004&rft_val_fmt=info:ofi/fmt:kev:mtx:journal&genre=article&sid=ProQ:ProQ%3Aiimpft&atitle=The+Development+of+a+Music+Therapy+Protocol%3A+A+

Werner, J., Wosch, T., & Gold, C. (2017). Effectiveness of group music therapy versus recreational group singing for depressive symptoms of elderly nursing home residents: pragmatic trial. *Aging Ment Health*, *21*(2 PG-147–155), 147–155. https://doi.org/10.1080/13607863.2015.1093599

Wilbur, S., Meyer, H. B., Baker, M. R., Smiarowski, K., Suarez, C. A., Ames, D., & Rubin, R. T. (2015). Dance for Veterans : A complementary health program for veterans with serious mental illness. *Arts & Health: International Journal for Research, Policy & Practice*, *7*(2 PG-96–108), 96–108. https://doi.org/10.1080/17533015.2015.1019701

Wilhelm, K., Gillis, I., Schubert, E., & Whittle, E. L. (2013). On a Blue Note: Depressed Peoples’ Reasons for Listening to Music. *Music & Medicine*, *5*(2 PG-76–83), 76–83. https://doi.org/10.1177/1943862113482143

Williams, E., Dingle, G. A., & Clift, S. (2018). A systematic review of mental health and wellbeing outcomes of group singing for adults with a mental health condition. *Eur J Public Health*, *28*(6 PG-1035–1042), 1035–1042. https://doi.org/10.1093/eurpub/cky115

Williams, G., & Dorow, L. G. (1983). Changes in complaints and non-complaints of a chronically depressed psychiatric patient as a function of an interrupted music/verbal feedback package. *Journal of Music Therapy*, *20*(3 PG-143–155), 143–155. https://doi.org/10.1093/jmt/20.3.143

Wittkower, E. D., & La Tendresse, J. D. (1955). Rehabilitation of chronic schizophrenics by a new method of occupational therapy. *British Journal of Medical Psychology*, *28*(1 PG-42–47), 42–47. Retrieved from http://www.embase.com/search/results?subaction=viewrecord&from=export&id=L281200161 NS  -

Witusik, A., & Pietras, T. (2019). Music therapy as a complementary form of therapy for mental disorders. *Pol Merkur Lekarski*, *47*(282 PG-240–243), 240–243. Retrieved from NS  -

Wolfgram, B. J. (1978). Music therapy for retarded adults with psychotic overlay: A day treatment approach. *Journal of Music Therapy*, *15*(4 PG-199–207), 199–207. Retrieved from http://search.ebscohost.com/login.aspx?direct=true&db=rih&AN=A63557&site=ehost-live&scope=site NS  -

Woodward, A. M. (2012). Arts-Based Practices in Regions Affected By War: An Overview of Where and How Arts-Based Practice are Applied and Studied in Countries Affected by War. *Voices: A World Forum for Music Therapy*, *12*(2 PG-NP), NP. Retrieved from https://search.proquest.com/docview/1370898147?accountid=11752 http://findit.library.jhu.edu/resolve?url_ver=Z39.88-2004&rft_val_fmt=info:ofi/fmt:kev:mtx:journal&genre=article&sid=ProQ:ProQ%253Aiimpft&atitle=Arts-Based+Practices+in+Regions+Affected+By+War

Xia, J., & Grant, T. J. (2009). Dance therapy for schizophrenia. *Cochrane Database Syst Rev*, (1 PG-Cd006868), Cd006868. https://doi.org/10.1002/14651858.CD006868.pub2

Yang, C. Y., Chen, C. H., Chu, H., Chen, W. C., Lee, T. Y., Chen, S. G., & Chou, K. R. (2012). The effect of music therapy on hospitalized psychiatric patients’ anxiety, finger temperature, and electroencephalography: a randomized clinical trial. *Biol Res Nurs*, *14*(2 PG-197–206), 197–206. https://doi.org/10.1177/1099800411406258

Yang, C. Y., Miao, N. F., Lee, T. Y., Tsai, J. C., Yang, H. L., Chen, W. C., … Chou, K. R. (2016). The effect of a researcher designated music intervention on hospitalised psychiatric patients with different levels of anxiety. *J Clin Nurs*, *25*(5-6 PG-777–87), 777–787. https://doi.org/10.1111/jocn.13098

Yang, W. J., Bai, Y. M., Qin, L., Xu, X. L., Bao, K. F., Xiao, J. L., & Ding, G. W. (2019). The effectiveness of music therapy for postpartum depression: A systematic review and meta-analysis. *Complement Ther Clin Pract*, *37*(PG-93-101), 93–101. https://doi.org/10.1016/j.ctcp.2019.09.002

Yoon, S., Verona, E., Schlauch, R., Schneider, S., & Rottenberg, J. (2019). Why do depressed people prefer sad music? *Emotion*, (PG-). https://doi.org/10.1037/emo0000573

Zagelbaum, V. N., & Rubino, M. A. (1991). Combined dance/movement, art, and music therapies with a developmentally delayed, psychiatric client in a day treatment setting. *The Arts in Psychotherapy*, *18*(2 PG-139–148), 139–148. https://doi.org/10.1016/0197-4556(91)90021-2

Zarate, R. (2016). Clinical Improvisation and its effect on Anxiety: A Multiple Single Subject Design. *Arts in Psychotherapy*, *48*(PG-46-53), 46–53. https://doi.org/10.1016/j.aip.2015.11.005

Zarate, R. (2016). The Social Architecture of Anxiety and Potential Role of Music Therapy. *Voices: A World Forum for Music Therapy*, *16*(1 PG-np), np. Retrieved from https://search.proquest.com/docview/1780078176?accountid=11752 http://findit.library.jhu.edu/resolve?url_ver=Z39.88-2004&rft_val_fmt=info:ofi/fmt:kev:mtx:journal&genre=article&sid=ProQ:ProQ%3Aiimpft&atitle=The+Social+Architecture+of+Anxiety+and+Potential+

Zarate, R. (2013). *The sounds of anxiety: A quantitative study of music therapy and anxiety*. ProQuest Information & Learning. Retrieved from http://search.ebscohost.com/login.aspx?direct=true&db=psyh&AN=2013-99060-232&site=ehost-live&scope=site NS  -

Zhao, K., Bai, Z. G., Bo, A., & Chi, I. (2016). A systematic review and meta-analysis of music therapy for the older adults with depression. *Int J Geriatr Psychiatry*, *31*(11 PG-1188–1198), 1188–1198. https://doi.org/10.1002/gps.4494

Zidani, M., Audet, J. S., Borgeat, F., Aardema, F., O’Connor, K. P., & Khazaal, Y. (2017). Augmentation of Psychotherapy through Alternative Preconscious Priming: A Case Series Exploring Effects on Residual Symptoms. *Front Psychiatry*, *8*(PG-8), 8. https://doi.org/10.3389/fpsyt.2017.00008
