# Supplementary material for: The Use of Music in the Treatment and Management of Serious Mental Illness: A Global Scoping Review of the Literature
Source: Front Psychol. 2021 Mar 31;12:649840. doi: 10.3389/fpsyg.2021.649840 (PMC8044514; doi:10.3389/fpsyg.2021.649840)
Supplement: Supplementary file 2 [file Data_Sheet_2.pdf]

Data Sheet 2: List of Measures

Standardized Questionnaires (n = 201)	
Stroop Test	Rey Auditory Verbal Learning Test (RAVLT)
Faux Pas Test	Verbal Fluency Test
Benton visual retention test (BVRT)	Somatoform Dissociation Questionnaire
Beck Anxiety Inventory (BAI)	Disability Status Scale (DSS-20)
Hamilton Anxiety Rating Scale (HAMA-A, HARS)	Category Fluency Test
Hamilton Depression Rating Scale (HRSD, HAM-D)	CVS (Comprehension, Vocabulary, Similarities)
State-Trait Anxiety Inventory (STAI)	Quality of Life Systematic Inventory (QLSI)
Perceived Stress Scale (PSS)	Quality of Life Scale (QLS)
Montgomery– Asberg Depression Rating Scale (MADRS)	State-Trait Anxiety Inventory for Children (STAI-C)
Zung Depression Scale	State-Trait Anger Expression Inventory-2 (STAXI)
Brief Psychiatric Rating Scale (BPRS)	Mini International Neuropsychological Interview (MINI)
Hospital Anxiety and Depression Scale (HADS)	Valutazione delle Abilità e Definizione degli Obiettivi (VADO)
Clinical Global Impression (CGI)	Nurse's Observation Scale for Inpatient Evaluation (NOSIE)
Global Assessment of Functioning (GAF)	Client Satisfaction Questionnaire
Semantic Differential Scale	PTSD Checklist Civilian (PCL-C)
WHO Disability Assessment Schedule 2.0 (WHODAS 2.0)	PTSD Stress Disorder Check List (PCL)
WHO Quality of Life Scale (WHOQOLBREF)	Posttraumatic Stress Diagnostic Scale (PDS)
WHO-5 Well-Being Index-5	Primary Care-PTSD screen (PC-PTSD-5)
Quality of Life Enjoyment and Satisfaction (Q-LES-Q)	PTSD-8
Quality of life survey RAND–36	Rosenberg Self Esteem Scale
Profile of Mood States (POMS)	UCLA Loneliness Scale
Positive and Negative Syndrome Scale (PANSS)	Index of Spouse Abuse (ISA)
Positive and Negative Affect Schedule (PANAS)	Social Avoidance and Distress Scale (SADS)
PTSD Checklist for DSM-5 (PCL 5)	Hopkins Symptom Check List
PTSD Symptom Scale Interview (PSS-I)	Quick Inventory of Depressive Symptomatology (QIDS)
Center for Epidemiologic Studies Depression Scale	Cornell Scale for Depression in Dementia (C-CSDD)
Beck Depression Inventory (BDI)	Edinburgh Postnatal Depression Scale (EPDS)
EuroQoL (EQ-5D-5L)	Revised Prenatal Distress Questionnaire (NuPDQ)

Brief Symptom Inventory (BSI)	Child Behavior Checklist (CBCL)
Wechsler Memory Scale (WMS)	The Cognitive Failures Questionnaire (CFQ)
Symbol Digit Modalities Test (SDMT)	Brief Negative Symptom Scale (BNSS)
Children's Depression Inventory (CDI)	Calgary Depression Scale for Schizophrenia (CDSS)
Rumination Reflection Questionnaire (RRQ)	Spirituality Index of Wellbeing (SIWB)
Scale for the Assessment of Negative Symptoms (SANS)	UKU (Side effects Rating) scale
Pittsburgh Sleep Quality Index (PSQI)	Life Skills Profile (LSP)
Neuropsychiatric Inventory (NPI)	Personal Questionnaire Rapid Scaling Technique (PQRST)
Inventory for the Assessment of Activation and Arousal modulation through Music (IAAM)	Toronto Alexithymia Scale (TAS–20)
Self-Concept Inventory (SKI, “SelbstkonzeptInventar”)	Multi-Dimensional Scale for Rating Psychiatric Patients (MSRPP)
Scale for the Assessment of Positive Symptoms (SAPS)	Thematic Apperception Test (TAT)
Characteristics of Auditory Hallucinations Questionnaire	Patient Health Questionnaire (PHQ-9) (PHQ-8)
Mini Sleep Questionnaire (MSQ)	Generalized Anxiety Disorder seven-item (GAD-7)
Technion Sleep Questionnaire	Oxford Happiness Questionnaire (OHQ)
Fast Assessment of Children’s Emotions (FACE)	Mini-Mental State Examination (MMSE)
Depression Adjective Checklist (DACL)	Brief Assessment of Cognition in Schizophrenia (BACS)
UCLA PTSD RI	Inventory of Interpersonal Problems (IIP)
Child’s Reaction to Traumatic Events Scale (CRTES)	Mindful Attention Awareness Scale (MAAS)
Screen for Child Anxiety Related Disorders (SCARED)	Sense of Coherence scale (SOC)
Speilberger State Anxiety Inventory	Harvard Trauma Questionnaire (HTQ)
Social Fear Scale	Geriatric Anxiety Scale (GAS)
Sociability Scale	Geriatric Depression Scale (GDS)
Core Self-Evaluation Scale (CSES)	Beck Hopelessness Scale (BHS)
State Hope Scale (SHS)	‘‘How Do You Feel?’’ questionnaire (HDYF)
Multidimensional Scale of Perceived Social Support (MSPSS)	Wake Forest Physician Trust Scale
Phenomenology of Consciousness Inventory (PCI)	Musical Interaction Rating Scale (MIR(S))
Visual Analog Scale (VAS)	Music Improvisation Rating (MIR)
Clinical Outcomes in Routine Evaluation (CORE)	McReynolds Ferguson Hospital Adjustment Scale (HAS)

Body Image Assessment (BIA)	Heidelberg State Inventory
Ten-Item Personality Inventory (TIPI)	Perception of Boundaries Scale (GO)
Satisfaction with Life Scale (SWLS)	Embodied Intersubjectivity Scale (EIS)
Body Self-Efficacy (BSE)	Giessen Complaint List for Children and Adolescents (GCL-CA)
Depression, Anxiety and Stress Scale (DASS-21)	Inventory of Life Quality in Children and Adolescents (ILC)
Heidelberger Befindlichkeitsskala (HBS)	Mental Health Continuum-Short Form (MHC-SF)
Therapeutic Reactance Scale	Negative Symptom Rating Scale
Social Interaction Anxiety Scale (SIAS)	Interest in Music Scale (IiM)
Interaction Anxiousness Scale (IAS)	Multidimensional Mood State Questionnaire
Audience Anxiousness Scale (AAS)	Warwick-Edinburgh Mental Well-Being Scale
Richards-Campbell Sleep Questionnaire (RCSQ)	Professional Care Team Burden Scale (PCBT)
Emotion Regulation of Others and Self (EROS)	Richmond Agitation and Sedation Scale (RAAS)
Persons related to others questionnaire-2 (PROQ-2)	Physical Activity Enjoyment Scale (PACES)
Multi-scale dissociation inventory (MDI)	Connor-Davidson Resilience Scale (CDR)
Altered States of Consciousness Scale (11D-ASC)	Texas Social Behaviour Inventory
Relationship Change Scale (RCS)	Revised Children's Manifest Anxiety Scale (RCMAS)
Subjective Well-Being in Patients with Schizophrenia under Neuroleptics (SWN-K)	Children’s Dissociative Experiences Scale and Post Traumatic Symptom Inventory (C-Des)
Quality of Life Enjoyment and Satisfaction Questionnaire (Q-LES-Q)	Child and Adolescent Functional Assessment Scale (CAFAS)
Kiddie Schedule for Affective Disorders and Schizophrenia (KIDDIESADS)	Adolescent Psychopathology Scale (APS)
Schedule for Affective Disorders and Schizophrenia (SADS)	Reynolds Adolescent Depression Scale, 2nd edition (RADS 2)
University of Rhode Island Change Assessment (URICA)	Adolescent Visual-Analog Recreational Music Making Assessment (A-VARMMA)
Thai Depression Inventory (TDI)	Dissociative Experiences Scale (DES)
Adolescent Anger Rating Scale (AARS)	Dissociative Experiences Scale Taxon (DES-T)
Positive State of Mind Scale (PSOMS)	Treatment Acceptability and Preference (TAP) questionnaire
Camarillo Dynamic Assessment Scales	Social Phobia Inventory for Children (SPAI-C)
Home and Community Social Behavior Scale (HCSBS)	Impact of Event Revised (IES-R)
Ekman test	Paced Auditory Serial Addition Test (PASAT)
Kessler 10 depression (distress) scale	Chart of interpersonal reactions in closed living environments (CIRCLE)
Clinical Dementia Rating (CDR)	Depression Mood Self-Report Inventory for Adolescence

Quality of Life in Alzheimer’s Disease (QOL-AD)	Lucas and Ludwick mood scale
Quality of Life Inventory (QOLI)	Anxiety Sensitivity Index-3 (ASI-3)
Group Climate Questionnaire (GCQ-S)	Subjective Units of Distress Scale (SUD)
Life Events Checklist (LEC-5)	Short Mood and Feelings Questionnaire (SMFQ)
ENRICHD Social Support Instrument (ESSI)	Strengths and Difficulties Questionnaire (SDQ)
Social Skills Improvement System (SSIS)	Pediatric Quality of Life Inventory 4.0 Generic Core Scale (PedsQL 4.0)
Behavioral and Symptom Identification Scale (BASIS-32)	Gießentest self-assessment (GTS)
Trauma Related Guild Inventory (TRGI)	Gießentest observer assessment (GTFm)
Scale of Body Connection (SBC)	Scales for mental health (SPG)
Difficulties in Emotion Regulation Scale (DERS)	Social Skills Assessment
Body Investment Scale	Snaith-Hamilton Pleasure Scale (SHAPS)
Ann Arbor Mental Status Scale	Barcelona Music Reward Questionnaire (BMRQ)
Jenkins Symptom Rating Sheet	Impact Message Inventory (IMI-R)
Lorr scale (general functioning)	Relationship Questionnaire (RQ)
Albany Behavioural Rating Scale	Fatigue Scale
Functional assessment of chronic illness therapy-spiritual well-being scale (FACIT-Sp-12)	
**Qualitative Methods (n = 35)**	
Qualitative description/analysis	Interpretative phenomenological analysis
Qualitative Assessment	Autoethnography
Qualitative Interview	Think-Aloud measure
Interview	Children's actions and thoughts
Semi-structured Interview	A six-dimensional framework
Anecdotal testimony	Description of music therapy
Focus Group	Compared drawings based on color, line and movement
Case Study	Description of patient's progress through vignettes of different phases of treatment
Observation	Comparison of narratives made with and without music
Psychiatrist analysis	Human Figure Drawing Test (HFDT)/Draw-A-Person (DAP) Test
Self-Report	Test for Creative Thinking - Drawing Production (TCT-DP)
Session record forms/post-therapy individual patient reports	Rorschach assessment
First-person recounting	Musical Experience Interview'

Thematic Analysis/Responses	Drop out from therapy (rate)
Facial, gestural, vocal or (para-)verbal expressions	Total and component costs of the interventions
Questionnaire not specified	Personal Activities of Daily Living (PADL)
Measure not specified	Songwriting Assessment for Hopelessness
Face scale (mood)	
**Custom Questionnaires (n = 13)**	
Likert Scale created	Patient Self Ratings with adjective list based on Semantic Differential Scale
Researchers developed own scales	Volwiler Body Movement Analysis (VBMA)
Total complaint verbalizations	Custom word associations (WA) questionnaire
Idiosyncratic Measure of Convictions in Thoughts	Custom assessment of the patient reaction questionnaire
Rhythmic Attunement Scale – Psychosis (RASP)	Index of Musical Experience (IME)
Modified Version of Interpersonal Behavior Inventory (IBI)	Elderly Information Form
Interpersonal Reaction Test (IPRT)	
**Biomarkers (n = 11)**	
Electromyography (EMG)	Finger Temperature
Cervical-trapezius electromyography (CT)	Pulse Rate/Heart Rate
Skin Conductance	Immediate Mood State
Skin temperature (ST)	fMRI
Electroencephalogram (EEG)	Brain activation of medial prefrotal cortex
Biomarkers	
**Physical Performance Tests (n = 11)**	
Evaluations of sleep	MACC (motility)
Physical Agility	Eyes Test
One Leg Stance test (OLS)	Continuous Performance Test-Identical Pairs (CPT-IP)
Timed Up and Go test (TUG)	Beery Visual–Motor Integration Test (VMI)
Ambulatory monitoring	Chu's Attention Test
Trail Making Test	
