# Supplementary material for: The Use of Music in the Treatment and Management of Serious Mental Illness: A Global Scoping Review of the Literature
Source: Front Psychol. 2021 Mar 31;12:649840. doi: 10.3389/fpsyg.2021.649840 (PMC8044514; doi:10.3389/fpsyg.2021.649840)
Supplement: Supplementary file 3 [file Data_Sheet_3.pdf]

Data Sheet 3: General Reporting Recommendations

Information	Sample Details/Questions
**Use Standardized Reporting Guidelines**	Use appropriate, existing reporting guidelines for study design (e.g., CONSORT for RCTs, STROBE for observational studies, CARE for case reports, etc.) See the Equator Network for details: https://www.equator-network.org/reporting-guidelines/
	Identify and utilize existing reporting guidelines for the conditions/populations of interest (e.g., Kaloupek et al., 2010)
	Identify and utilize the reporting guidelines for the types of data collected as part of the study (e.g., eMOOD (Faurholt-Jepsen et al., 2019), fMRI (Poldrack et al., 2008))
**Population Under Study**	Demographics: race/ethnicity; gender; age; condition/diagnosis (if applicable); geographic region; group (if applicable; e.g., veteran, high school football team, new mothers, etc.). May also include education, socioeconomic status, language.
	How were participants selected for inclusion in the program or study? (If diagnosis was an inclusion criterion, was diagnosis confirmed? If so, how?)
	How long have participants lived with the condition under study?
	Have participants experienced any previous failed therapies?
**Development of Project**	Is this project/program based on an existing theory or disciplinary framework? (If so, describe/cite.)
	Had this music-inclusive intervention been previously developed? If so, by whom?
	Had this music-inclusive intervention been previously evaluated? If so, by whom? What were the results?
	Alternatively, was this a bespoke music engagement designed specifically for a study / research endeavor? If so, who designed it? based on what research/literature?
**Facilitator Information**	Who facilitated the music-based aspects of this program/intervention? What were their credentials related to this involvement?
	What were the facilitator's precise roles? (How were they engaged with the participants and/or the program process--at each stage of the intervention?) Note that a facilitator's role or particular engagement often changes throughout a program/study; all stages and roles should be reported)
**Setting**	What was the setting for the program/intervention? High levels of description are valuable for determining potential effects of environment on outcomes.
	Location (clinic, community center, school, faith setting, outdoors, etc.); description (size of room, layout, spaciousness, windows, current/dated decor, etc.); privacy level; etc.
**Dose and Duration**	How much time during each session was the participant engaged in the music-based activity?
	How many sessions were held? How often? Over how many days/weeks/months/years?
**Participant Choice, Autonomy**	Did the program/study involve music as one among many activities "on the menu"? (For example, did patients select a music activity from a variety of options (e.g., yoga, meditation, horseback riding, etc.)? If so, note any other options available, and what factors may have played into participants' choices.
	Did participants choose how long during each session to engage in the activity?
	Did participants choose how many sessions to attend?
