# Supplementary material for: The Use of Music in the Treatment and Management of Serious Mental Illness: A Global Scoping Review of the Literature
Source: Front Psychol. 2021 Mar 31;12:649840. doi: 10.3389/fpsyg.2021.649840 (PMC8044514; doi:10.3389/fpsyg.2021.649840)
Supplement: Supplementary file 4 [file Data_Sheet_4.pdf]

Data Sheet 4: Reporting Recommendations to Produce Detailed Descriptions of Music-Based Activities/Engagements

Intervention Detail	Sample Details/Questions
**Instrument**	Were participants playing an instrument(s)? Report which, with as much specificity as possible.<br>At the least, note whether it was wind instrument, percussive instrument such as piano or vibraphone, drum(s), drum set, stringed instrument with a bow, stringed instrument without a bow, etc. (Different instruments entail varied physical movements, which can affect outcomes.)
**Participant Impressions of Activity**	As possible, report on participants' impression of the activity before participating. (If a participant was already interested in the instrument/activity before beginning, or if they had a generally positive impression of the instrument/activity, these perceptions could affect outcomes.)
**Singing Details**	Report whether participant(s) were:<br>• Singing alone<br>• Singing with a facilitator/teacher/therapist<br>• Singing in a small/intimate group (e.g., group therapy or music class)<br>• Singing in a large, formal group (e.g., choir)<br>• Singing in a large, public group (e.g., at a concert or church)<br>• Singing acapella<br>• Singing to live music (document what the live music consisted of, such as a pianist, guitarist, other instrumentalist, group of musicians (e.g., band/orchestra) etc.<br>• Singing to recorded music (document how participants listened; e.g., on headphones, speakers, iPhone, etc.)
**Listening Details**	Report whether participants were:<br>• listening to live music alone or in an intimate group<br>• listening to live music in a venue or with a larger/public audience<br>• listening to recorded music alone with headphones<br>• listening to recorded music alone on speakers<br>• listening to recorded music in a small/intimate group<br>• listening to recorded music with a large group (such as in a faith community, at a concert, etc.)
**Dancing/Movement Details**	Report whether participants were:<br>• Dancing alone<br>• Dancing with a facilitator/teacher/therapist<br>• Dancing in a small/intimate group (e.g., dance therapy session)<br>• Dancing in a larger formal group (e.g., dance company or class)<br>• Dancing in a large public group (e.g., dancing at a public concert)<br>• Moving to music but not in a way the participant describes as "dance"<br>• Participating in exercise that follows music or its beat but is not described as "dance" (e.g., aerobics), etc.
**Lyric/Songwriting Details**	Report whether participants were:<br>• Writing song lyrics alone<br>• Writing song lyrics with a facilitator/teacher/therapist<br>• Writing song lyrics with a small/intimate group (e.g., group therapy)<br>• Writing song lyrics to live music (describe)<br>• Writing song lyrics to recorded music (describe)<br>• Writing song lyrics while also composing one's own music (with an instrument? acapella? other?)<br>• Writing song lyrics without music
**Genre Details**	• What genre/style was the music?<br>• How/why was the genre selected? By whom?<br>• Who identified the genre as documented in the report? (See "General," below)
**Participant Choice/Autonomy**	• Report how and when decisions were made throughout both the planning and implementation of the intervention, with an emphasis on when and to what extent participant choice was involved.

	• Who decided to create, begin, research, or evaluate the music-inclusive program/intervention? Based on what factors?<br>• Who chose which music-based activities would be incorporated? Based on what factors?<br>• If activities changed throughout each session or throughout the program, who determined which activity would be engaged and when? Based on what factors?<br>• Who chose the genre of music that would be heard, played, sung, composed?<br>• Who made specific song choices within that genre?<br>• Who selected the volume?<br>• Who made decisions between headphones and speakers?<br>• Who decided when to start or stop a given song, activity, etc.? On what was this decision based?<br>• Who determined dosage/duration? based on what criteria/factors?
**General**	Regardless of specific activities, always report on the following:<br>• Genre, including who selected it *and* who identified it as the listed genre. (For example, did the participant refer to the music as "classical," or did they choose a piece of music, and the researcher or facilitator later labeled it as "classical"?)<br>• How the music was heard (speakers, live acoustic, live amplified, headphones, iPhone or similar device without headphones, etc.)<br>• The quality of the sound, whether in a live space (was it professionally amplified/mixed?) or on a recording (was it professionally recorded?). Document any issues with sound quality (for example, if a recording is garbled, or if a live singer is difficult to hear in the room). Such issues can affect outcomes.<br>• How facilitators/researchers ensured that participants could hear the music well (or as well as they wished)<br>• Who made choices related to: 1) When to start/stop the music or the music-based activity; 2) Volume selection; 3) When to switch from one dynamic to another, one activity to another, one genre to another, etc. (See "Participant Choice/Autonomy)
**Modifications**	• If the program/intervention allowed for tailoring (such as in response to Participant Choice/Autonomy), document how the initial protocol was adapted or modified during implementation. For example, at what stages of the intervention were modifications made? Who initiated them (participant or facilitator)? In what ways did these modifications affect other aspects of the intervention?
